# Association of the Schizophrenia Risk Locus rs10866912 with Cerebral Volumes, Diet, and Psychosis in the UK Biobank

**DOI:** 10.3390/genes17101207

**Published:** 2026-09-29

**Authors:** Lauren Jankowski, Sathish Periyasamy, Pierre Youssef, Sujit John, Katherine L. Forthman, Rayus Kuplicki, Jean Giacomotto, Heather Smith, Duncan McLean, Vijaya Raghavan, Rangaswamy Thara, Martin Paulus, Bryan Mowry

**Affiliations:** 1 Queensland Brain Institute, The University of Queensland, Brisbane, QLD 4072, Australia; laurenjankowski1@gmail.com (L.J.); sathish.periyasamy@monash.edu (S.P.); j.giacomotto@uq.edu.au (J.G.); h.smith8@uq.edu.au (H.S.); duncan_mclean@qcmhr.uq.edu.au (D.M.); 2 Queensland Centre for Mental Health Research, West Moreton Hospital and Health Service, Brisbane, QLD 4076, Australia; 3 QIMR Berghofer Medical Research Institute, Brisbane, QLD 4006, Australia; pierre.youssef@qimrberghofer.edu.au; 4 Schizophrenia Research Foundation, Chennai 600101, India; vijayaraghavan@scarfindia.org (V.R.); thara@scarfindia.org (R.T.); 5 Laureate Institute for Brain Research, Tulsa, OK 74136, USA; kforthman@laureateinstitute.org (K.L.F.); rkuplicki@laureateinstitute.org (R.K.); mpaulus@laureateinstitute.org (M.P.)

**Keywords:** genetics, genomics, schizophrenia, neuroimaging, nutrition, gene–environment interaction

## Abstract

Background: Leading schizophrenia GWAS signals require evaluation of their pathological significance across diverse ancestries. rs10866912 reached genome-wide significance in our Indian ancestry GWAS of schizophrenia; its risk allele reduces expression of NAPRT, the rate-limiting enzyme in the brain’s principal NAD+-synthesis pathway, giving a single mechanistic basis for testing associations with brain structure, dietary niacin intake, and psychosis risk. We evaluated this SNP in a large European ancestry population for associations with regional brain volumes, niacin-rich food consumption, and psychotic disorder diagnosis. Methods: Of approximately 500,000 UK Biobank participants, 36,492 met study criteria of European ancestry, brain MRI examination, and dietary questionnaire completion. Risk-allele count was regressed on each of 174 phenotypes—139 regional gray matter volumes, 26 psychological and 9 dietary variables—with age, sex, and BMI as covariates. Volumes were log-transformed and adjusted for total gray matter volume by residualization, and Bonferroni and false discovery rate corrections were applied within each domain. Results: Risk-allele count was negatively associated with gray matter volume in the left pallidum (β = −0.0143, *p* = 0.00016; Bonferroni-significant) and left caudate (β = −0.0142, *p* = 0.00038; FDR-significant), with the contralateral homologues in the same direction at nominal significance. No dietary or psychological variable survived correction. Nominally significant associations included increased wheat consumption and fewer years spent vegetarian, both implying higher dietary niacin. The association with a diagnosis of psychotic disorder, nominally significant in linear regression, was not significant when the binary outcome was re-examined by logistic regression (OR = 1.36, 95% CI 0.997–1.85, *p* = 0.053). Conclusions: The risk allele is associated with reduced pallidal and caudate gray matter volume in a large general-population sample, in a direction consistent with observations in unmedicated patients once antipsychotic effects are allowed for. The dietary and psychiatric associations are exploratory and warrant further study of the relationship between this locus, circulating niacin, and psychotic disorder.

## 1. Introduction

A recent GWAS of 3092 Tamil individuals with schizophrenia identified a genome-wide significant SNP (rs10866912) on chromosome 8q24.3, with C being the protective allele and A the risk allele. Further analysis identified that this SNP influences NAPRT (nicotinate phosphoribosyltransferase), a gene that produces a rate-limiting enzyme in nicotinic acid (niacin) metabolism and is involved in the Preiss–Handler pathway. This pathway plays a crucial role in the biosynthesis of nicotinamide adenine dinucleotide (NAD+), a coenzyme essential for many cellular processes, including energy metabolism, DNA repair, and cell signaling [1,2,3]. In the context of brain function, this pathway’s significance is underscored by NAD+’s involvement in maintaining mitochondrial health, supporting neuronal energy demands, and protecting against neurodegeneration. Given the brain’s high energy consumption and the vulnerability of neurons to metabolic stress, the efficient synthesis of NAD+ through the Preiss–Handler pathway has been hypothesized to have profound implications for cognitive functions, neural resilience, and the overall maintenance of neural health [4]. Periyasamy et al. [5] found that Indian lymphoblastoid cell lines homozygous for the protective allele (genotype: CC) at this SNP displayed nearly double the NAPRT mRNA expression compared to cell lines homozygous for the risk allele (genotype: AA). As NAPRT plays a crucial role in the Preiss–Handler pathway, this raises the question of whether decreased expression of NAPRT causes downregulation of this pathway. While there are three different metabolic pathways that can produce NAD+, the Preiss–Handler pathway is thought to be the main supply for NAD+ in the brain [3].

A second pathway that results in the production of NAD+ is the de novo biosynthesis pathway, which converts tryptophan from the diet into NAD+. Researchers speculate that a vegetarian diet, which may be deficient in tryptophan, could be a contributing risk factor for the presence of schizophrenia in subjects with the risk alleles, as the Preiss–Handler pathway would theoretically be downregulated due to decreased expression of NAPRT. A slight increase in vegetarianism was identified in the risk allele cohort, but more studies are needed to confirm any relationship [5].

This same study also found that a partial loss of function of NAPRT in animal models, e.g., zebrafish, resulted in abnormal brain development, specifically holoprosencephaly, where the two hemispheres in the brain do not divide completely. While this does not directly tie rs10866912 to abnormal brain development, it suggests a potential relationship between this SNP, its effect on NAPRT expression, and the resultant effects on the Preiss–Handler pathway and potentially brain development. It is hypothesized then that the under-expression of NAPRT may result in decreased NAD production in the brain, which has been shown to cause neurodegenerative disorders with psychotic-like symptoms [6]. The presence of NAD is also thought to in part regulate oxidative stress; thus, the downregulation of NAPRT and potential decreased production of NAD may result in increased oxidative stress [7]. Patients with schizophrenia have been shown to display increased levels of oxidative stress, and while it is not believed to be a cause of the disease, oxidative stress is thought to be a factor in the poor prognosis and declining course of schizophrenia [8].

The Minor Allele Frequency (MAF) of this SNP has undergone natural selection over time, with the risk allele of our top SNP, rs10866912, declining in frequency from Africa (97%) to India (72%) to Europe (43%) [9]. Due to the high frequency of the risk allele in South Asia, we were able to detect this variant with greater statistical power (*p* < 5 × 10^−8^) than in Europeans (*p* < 7.5 × 10^−4^) [5].

In a U.S. study of 500 diagnostically interviewed individuals with depression [10], MRI imaging was conducted, and neuropsychological and dietary parameters were assessed. This study found that 225 subjects had one risk allele at rs10866912, 128 had two risk alleles, and the remaining 127 subjects had no risk alleles [11]. Individuals with the risk allele showed statistically significant volume reduction in the temporal lobes, as well as lower social ability and cognitive ability, when compared to individuals without the risk allele. Additionally, individuals with the risk allele were more likely to report eating eggs, which are niacin-rich, than their non-risk allele counterparts, suggesting a possible compensation for the downregulation of the Preiss–Handler pathway. It is hypothesized that an unknown mechanism results in increased consumption of niacin-rich foods in an attempt to counter the suspected downregulation of the Preiss–Handler pathway. Given the small sample size of this study (*n* = 480), we resolved to evaluate this finding in a significantly larger sample.

Taken together, the rationale for testing rs10866912 against three distinct outcome domains—regional brain volume, dietary choice, and psychotic disorder diagnosis—follows a single, biologically coherent chain rather than an unrelated set of associations. First, this SNP reached genome-wide significance in our GWAS of schizophrenia in a South Indian population [5], so testing its association with a psychotic disorder diagnosis in an independent, ethnically distinct cohort constitutes a direct test of generalizability. Second, the risk allele reduces NAPRT expression and, in turn, flux through the Preiss–Handler pathway believed to be the principal source of brain NAD+, providing a specific and biologically plausible route by which this SNP could affect brain structure and function rather than a purely statistical association. Third, because partial NAPRT loss-of-function impairs brain development in zebrafish and NAD+ deficiency is linked to neurodegeneration [5,6] and because a smaller U.S. study found the same risk allele associated with reduced temporal lobe volume [11], we hypothesized a parallel relationship with regional gray matter volume in a substantially larger sample. Fourth, because impaired Preiss–Handler flux might be offset by increased dietary niacin intake, and the same U.S. study found risk-allele carriers more likely to report eating niacin-rich eggs [11], we tested whether the risk allele predicted a dietary preference for niacin-rich foods. Each outcome domain examined here therefore follows directly from the same underlying NAPRT/NAD+ mechanism.

In the UK Biobank population, we sought to (1) determine the relative frequencies of the risk and protective alleles at rs10866912; (2) identify potential associations between diet and the risk allele; (3) replicate the risk allele association with lesser regional brain volumes; and (4) test the risk allele association with a psychotic disorder diagnosis.

## 2. Materials and Methods

### 2.1. Sample Selection

The UK Biobank (UKBB) cohort is predominantly composed of individuals of European ancestry, aged 40 to 69 at the time of recruitment (2006–2010). Analyses were conducted under UK Biobank project number 64593. Participants completed touch-screen questionnaires relating to sociodemographic, physical and mental health history, and lifestyle. Various physical examinations and measurements were also performed on subsets of individuals. Participants provided electronic consent for use of their genomic data, self-completed questionnaire answers, and physical/physiological data measurements. The number of individuals who completed the psychological questionnaire was much smaller than the number used for the diet and brain volume analyses. Response rates for the psychological variables ranged from approximately 7000 to 25,000 of the total 36,492 samples.

Individuals who had undergone T1-weighted structural MRI and had recorded regional gray matter volumes (data-fields 25782-25920) were selected. These regional volumes were derived by the UK Biobank imaging pipeline (FMRIB’s Biobank Pipeline), in which gray matter partial volume estimates from FAST (FMRIB’s Automated Segmentation Tool) are averaged within 139 regions of interest defined by the Harvard–Oxford cortical and subcortical atlases and the Diedrichsen cerebellar atlas (UK Biobank Category 1101). Of these, individuals who had provided a response to UKBB data-field 6144 (“Which of the following do you never eat?” with choices of “eggs or foods containing eggs”, “dairy products”, “wheat products”, “sugar or food/drinks containing sugar”, “I eat all of the above”, and “prefer not to answer”) were retained. Covariates of age, sex, and BMI were selected, and any individuals without one or more of these variables were removed. Psychological and other dietary variables that were added later in the analysis were not used as selection criteria.

See Supplementary Material for Pre- and post-imputation details.

### 2.2. Analysis

Prior to performing linear regression, several variables were recoded as binary. While the data for gray matter volume in various brain regions were quantitative, many of the dietary and psychological variables were categorical or binary.

In all analyses, the number of risk alleles was the dependent variable, and each phenotype was entered as a predictor, together with age, sex and body mass index. Binary and categorical phenotypes therefore enter the models as predictors rather than as outcomes, so no distributional assumption is placed upon them; for a binary predictor the coefficient is the difference in mean risk-allele count between the two groups, and for an ordered categorical predictor it is the change in mean risk-allele count per category. The single exception is diagnosis of a psychotic disorder, which was additionally re-examined with the roles reversed, treating diagnosis as a binary outcome and risk-allele count as the predictor, by logistic regression with age, sex and body mass index as covariates, since that is the form in which a clinical outcome is conventionally reported.

The number of risk alleles (A) possessed by each individual at rs10866912 was calculated (using R-4.2) and added to a file containing the brain volume, dietary, and psychological variables for each individual, including the covariates of age, sex, and BMI. The distribution of the number of risk alleles was calculated and visualized in a histogram.

All non-binary variables were assessed for a normal distribution prior to regression, and if a variable was skewed, it was transformed using the optLogTransform() function from the R package optLog [12]. The package is not versioned; the version used corresponds to the repository state at its last update on 1 August 2019. Models were fitted both with and without covariates. The ‘summary’ function was used on each model to identify the intercept, beta value, *p*-value, and R2 value. The R2 difference between the linear model fitted with covariates and that without was calculated.

The negative log10 of the *p*-value resulting from the hypothesis test, with a null hypothesis of no linear relationship between the number of risk alleles and the variable of interest, was used to generate Phenome-wide association plots to show the significance of the relationship between the number of risk alleles and the phenotype of interest. Three levels of significance were assessed: a nominal *p*-value of 0.05, a False Discovery Rate (FDR) adjusted *p*-value of 0.05, and a Bonferroni-adjusted *p*-value of 0.05. These two higher levels of significance were deemed the threshold for meaningful significance.

Post hoc minimum-detectable-effect-size calculations (Discussion; Figure S13) were computed from the formula given in Section 4 and can be verified independently; an earlier version of these calculations was produced with AI assistance.

### 2.3. Statistical Adjustments

#### 2.3.1. Adjustment for Total Gray Matter Volume

We adjusted regional volumes for global gray matter volume using the residualization method, which is the standard approach in neuroimaging research for controlling for differences in overall brain size while preserving individual region-specific variance. A T1-derived intracranial volume was not available in the analysis dataset, so total gray matter volume (TGMV) was used as the size covariate, calculated as the sum of all 139 gray matter volumes for each individual (mean = 615,593.7 mm^3^, SD = 57,360.4 mm^3^). This is an adjustment for global gray matter volume and not for intracranial volume: because it excludes white matter and cerebrospinal fluid, it does not index cranial capacity, and it should not be read as a complete adjustment for head size, since residual head-size effects cannot be excluded. To adjust individual brain volumes for TGMV while avoiding multicollinearity issues inherent in direct covariate inclusion (wherein both component volumes and their sum appear in the same regression), we employed the residualization method recommended in the neuroimaging literature [13].

For each brain region, we calculated adjusted volumes as residuals from the regression of log-transformed volume on TGMV. This approach produces TGMV-adjusted volumes that are mathematically uncorrelated with TGMV, effectively removing the confounding effect of overall brain size while preserving region-specific neurobiological associations with genotype. The TGMV-adjusted volumes were then entered as predictors in the regression model, where nA (number of rs10866912 risk alleles) was regressed on the TGMV-adjusted volume plus age, sex, and BMI.

#### 2.3.2. Multiple Testing Corrections

To maintain statistical rigour while accounting for multiple comparisons, three levels of significance were applied to all analyses.

Nominal significance (α = 0.05) denotes uncorrected *p*-values, used to identify associations of potential interest for hypothesis generation, particularly in the exploratory psychiatric and dietary domains.

False discovery rate (FDR) correction was applied within each analytical domain using the Benjamini–Hochberg procedure, which controls the expected proportion of false positives among rejected hypotheses.

Bonferroni correction was applied by dividing α = 0.05 by the number of tests within each domain: brain imaging, α = 0.05/139 = 3.60 × 10^−4^; psychiatric and psychological variables, α = 0.05/26 = 1.92 × 10^−3^; dietary variables, α = 0.05/9 = 5.56 × 10^−3^.

Corrections were applied separately within each domain rather than across all 174 phenotypes because each domain represents a distinct a priori hypothesis with its own biological rationale within the NAD+ biosynthesis pathway. rs10866912 was pre-specified on the basis of genome-wide significant discovery in an independent Indian cohort [5] and a validated functional effect on NAPRT expression, rather than selected from an undirected screen of mechanistically uncharacterised variants; this hypothesis-driven design reduces the multiple-testing burden relative to a conventional candidate-gene screen. All corrections were implemented in R (version 4.2), using p.adjust() with method = “BH” for FDR and explicit per-domain thresholds for Bonferroni.

### 2.4. Use of AI-Assisted Tools

The study was conceived, designed, analyzed, and written up by the authors over a period of several years. Claude (Anthropic) was used during final preparation and revision of the manuscript, as an interactive assistant under continuous author direction, to draft text and analysis scripts, with all output checked against the data and against primary sources. The study design, the interpretation of the results and the conclusions are the authors’ own.

## 3. Results

See Supplementary Material for details of sample selection, pre- and post-imputation data processing.

### 3.1. Distribution of Risk Alleles Within the UKBB European Subset

**Figure 1:**
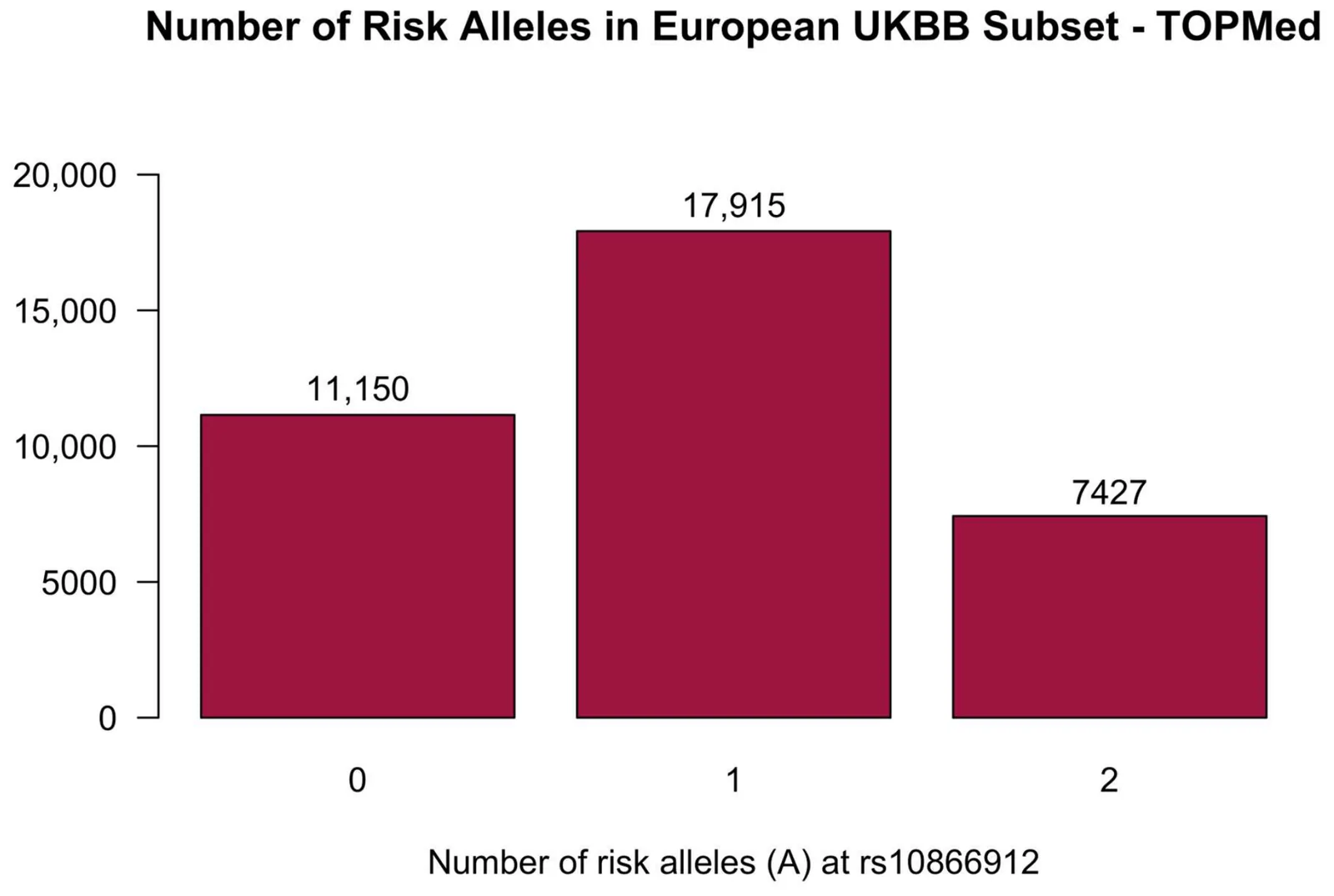
Distribution of risk alleles in the European UKBB subset. Genotypes were imputed using the TOPMed Imputation Server and the TOPMed r2 reference panel.

Within the TOPMed-imputed dataset, 49% of individuals were found to be heterozygous at this SNP, 31% homozygous for the protective allele (C), and 20% homozygous for the risk allele (A). A histogram displaying the specific distribution for the imputed dataset is shown in Figure 1.

### 3.2. Linear Regression

- Gray matter volumes

The gray matter volume within 139 brain regions was assessed. The gray matter volume within eight brain regions was found to show statistically significant correlation with the number of risk alleles possessed by an individual when covariates were included: the right cerebellum (lobule X), right and left pallidum, right and left caudate, left planum polare, anterior right cingulate gyrus, and left intracalcarine cortex. All regions except the right cerebellum (X) showed a decrease in gray matter volume as the number of risk alleles increased, whereas the gray matter volume in the right cerebellum increased. All except the right cerebellum (X) also reached statistical significance when covariates were not included in the regression models. The *p*-value, beta value, and intercept of the linear model with covariates for each brain region that exceeded the nominal *p*-value can be seen in Table S6.

**Figure 2:**
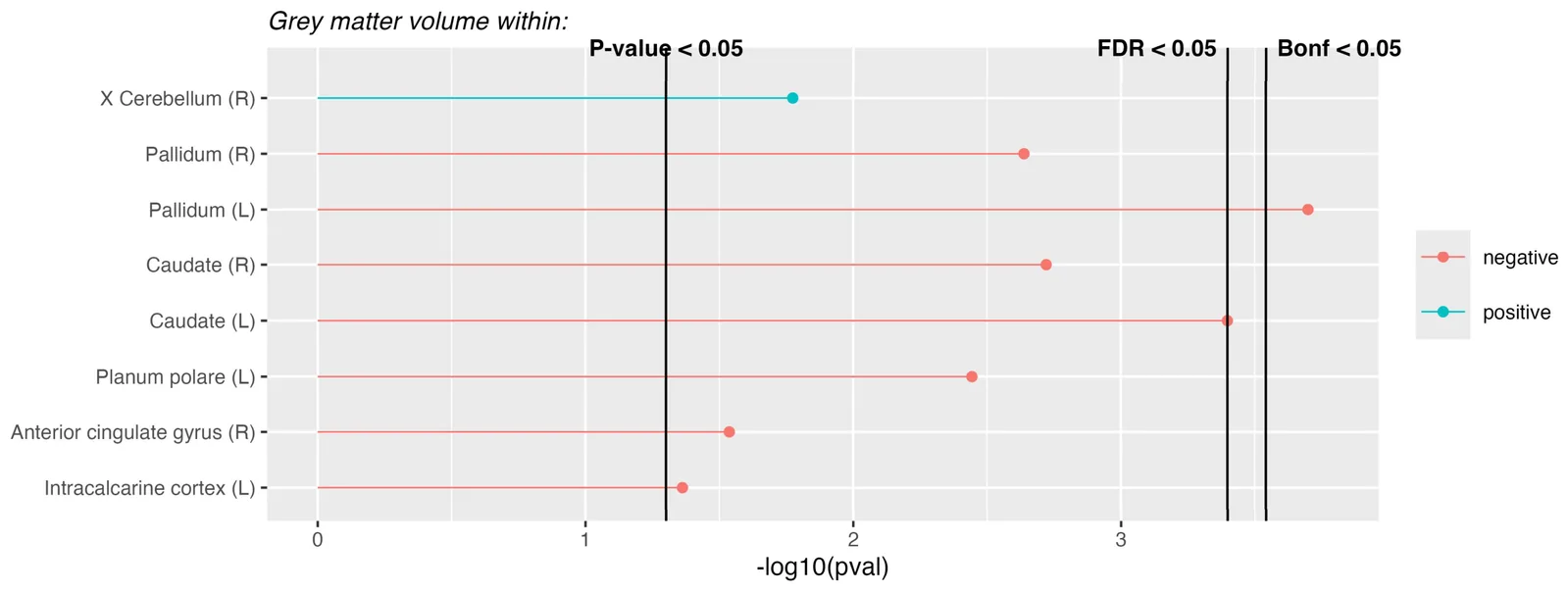
Significant correlations between changes in gray matter volume and number of risk alleles at rs10866912. Phenome-wide association plot showing −log10 *p*-values resulting from linear association tests on the gray matter volume within various brain regions and number of risk alleles in the TOPMed-imputed data.

Two brain regions reached the higher thresholds for significance. The association between the number of risk alleles at rs10866912 and gray matter volume within the left pallidum reached Bonferroni-level significance. The gray matter volume within the left caudate reached FDR-level significance. Figure 2 shows a Phenome-wide association plot with the direction of correlation and −log10 of the *p*-value of each variable.

- Analysis Adjusted for Total Gray Matter Volume

To account for individual differences in overall brain size, the above analyses were repeated using regional volumes adjusted for total gray matter volume (TGMV) via residualization. Results remained robust following TGMV adjustment. The left pallidum association maintained Bonferroni-level significance (β = −0.0143, 95% CI: −0.0218 to −0.0069, *p* = 0.00016), and the left caudate association maintained FDR-level significance (β = −0.0142, 95% CI: −0.0220 to −0.0063, *p* = 0.00038). Effect sizes and statistical significance were preserved, with minimal change from the original unadjusted analysis (Table 1). Detailed results for all 139 brain regions using TGMV-adjusted volumes are presented in Table S10. Associations in the contralateral homologues were in the same direction and nominally significant (right pallidum β = −0.0119, *p* = 0.0019; right caudate β = −0.0125, *p* = 0.0021; Table S10).

- Dietary choices

**Figure 3:**
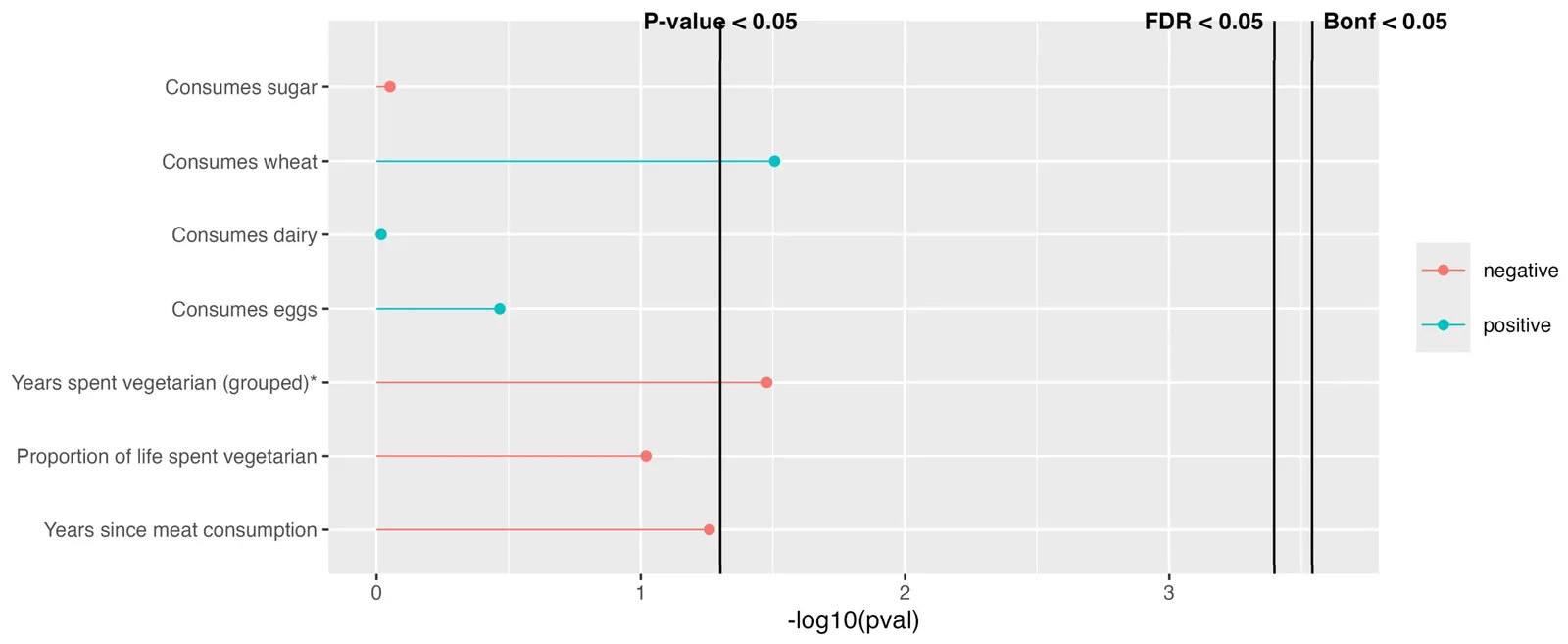
Significant correlations between dietary choices and number of risk alleles at rs10866912. Phenome-wide association plot showing −log10 *p*-values resulting from linear association tests on dietary variables and number of risk alleles in the TOPMed-imputed data. Years spent vegetarian is grouped as follows: 0–vegetarian for less than 20 years; 1–vegetarian for 20+ years but not a lifelong vegetarian; 2–lifelong vegetarian.

When covariates were included in the linear regression, the only variables reaching nominal significance were “consumption of wheat” (positive correlation with number of risk alleles) at rs10866912 and “years spent vegetarian” (negative correlation with number of risk alleles at rs10866912). Table S7 summarizes the linear regression models for each dietary variable, as well as the brain region and psychological variables. Figure 3 shows a Phenome-wide association plot with the direction of correlation and -log10 of the *p*-value of each variable.

- Psychological variables

**Figure 4:**
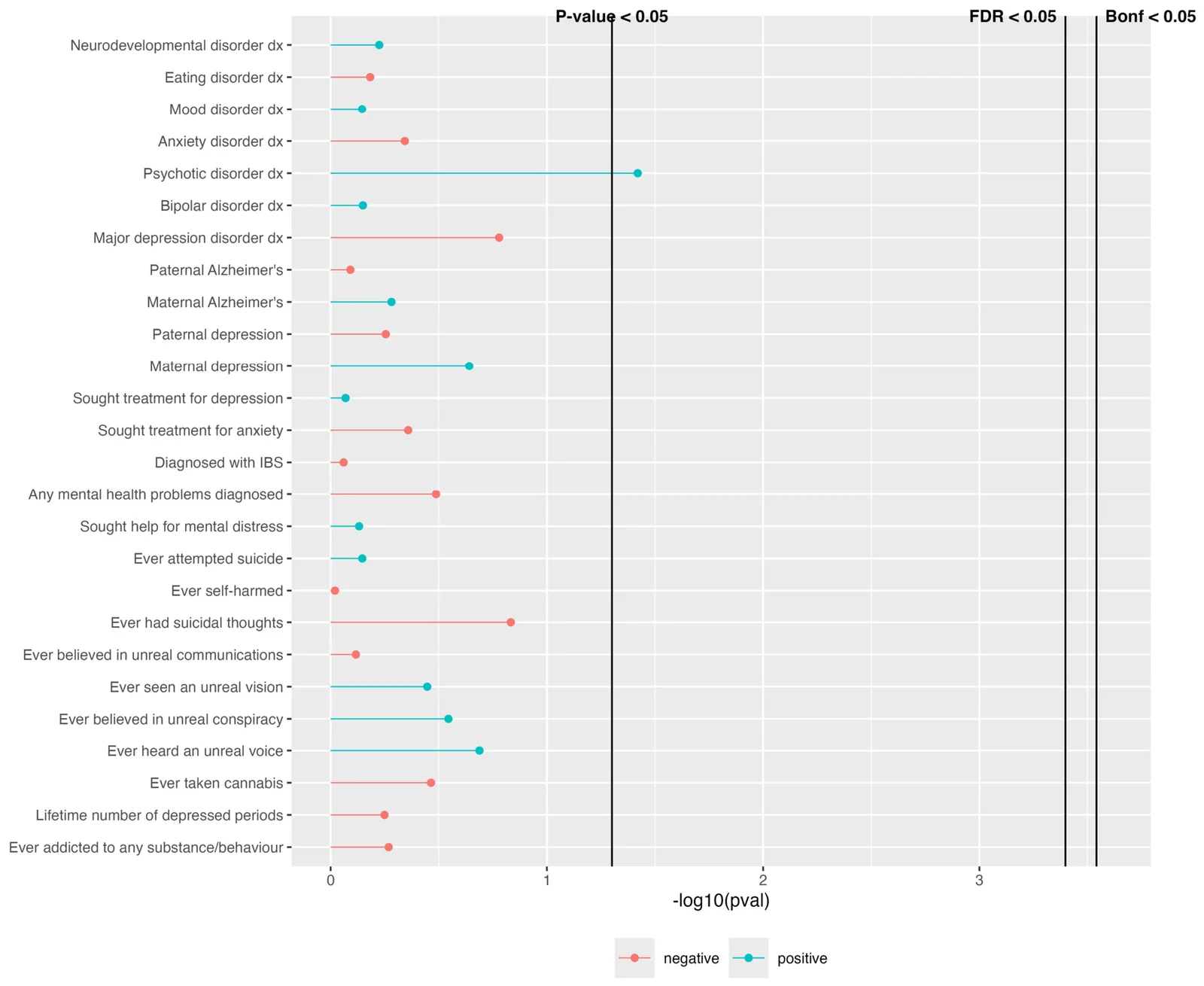
Significant correlations between psychological variables and number of risk alleles at rs10866912. Phenome-wide association plot showing −log10 *p*-values resulting from linear association tests on psychological variables and number of risk alleles in the TOPMed-imputed data.

Covariates were included in the linear regression for the psychology- and psychiatry-related variables. Of the 26 variables assessed, only a diagnosis of a psychotic disorder was found to demonstrate a statistically significant level of correlation (*p* < 0.05) with the number of risk alleles. The presence of a psychotic disorder positively correlated with the number of risk alleles at rs10866912. Because psychotic disorder diagnosis is a binary outcome, this association was re-examined using logistic regression with age, sex, and BMI as covariates (glm, binomial family). Each additional risk allele at rs10866912 was associated with increased odds of psychotic disorder diagnosis, but the association was not statistically significant (OR = 1.358, 95% CI: 0.997–1.849, *p* = 0.0526). Figure 4 shows a Phenome-wide association plot with the statistical significance and correlation direction of each variable assessed.

## 4. Discussion

In this study, we sought to understand the genetic and environmental factors contributing to schizophrenia, focusing on the association between a specific SNP, rs10866912, and its impact on *NAPRT* gene expression and the Preiss–Handler pathway. Our comprehensive analysis within the UK Biobank population aimed to explore how this genetic variation, in conjunction with dietary habits, affects brain structure and function, potentially influencing the risk and progression of schizophrenia. Our findings reveal that the presence of the risk allele (A) at rs10866912 is significantly associated with decreased gray matter volume in specific brain regions, including the left pallidum and caudate, areas critical for cognitive and emotional processing. In addition, we observed a correlation between the risk allele and dietary choices, with increased consumption of wheat and fewer years spent as a vegetarian showing nominally significant relationships with the presence of the risk allele. Finally, our analysis examined the relationship between the number of risk alleles and the diagnosis of psychotic disorders, highlighting the potential role of this genetic variant in contributing to the etiology of schizophrenia. Using logistic regression with age, sex, and BMI as covariates, each additional risk allele at rs10866912 was associated with increased odds of psychotic disorder diagnosis, but the association was not statistically significant (OR = 1.358, 95% CI: 0.997–1.849, *p* = 0.0526). Taken together, these results suggest that the A allele at rs10866912 may contribute to schizophrenia’s pathophysiology by affecting brain structure and function, potentially mediated through dietary influences and the downregulation of the Preiss–Handler pathway.

In our allele-specific analysis, we found that as the gray matter volume in the pallidum and caudate decreases, the number of risk alleles increases, suggesting a notable relationship between gray matter volume in certain brain regions and this SNP. Specifically, the association between the number of risk alleles at rs10866912 and gray matter volume within the left pallidum reached Bonferroni-level significance, and that within the left caudate reached FDR-level significance. Because the imaging sample included 80 individuals with a psychotic disorder diagnosis, in whom antipsychotic exposure could plausibly alter striatal volume, we repeated the analysis excluding them. The associations were essentially unchanged (left pallidum β = −0.0142, 95% CI −0.0217 to −0.0068, *p* = 0.00018; left caudate β = −0.0143, 95% CI −0.0221 to −0.0065, *p* = 0.00032; *n* = 36,412), indicating that the relationship reflects variation across the general population rather than the diagnosed subgroup.

The pallidum and caudate are part of the basal ganglia, which are responsible for voluntary motor movements and play a role in learning, cognition, emotion, and formation of habits. We can speculate that the decreased volume of the left caudate and left pallidum with increased number of rs10866912 risk alleles in this large sample of controls reflects a specific effect of this SNP on brain morphology; no implications for psychopathology can be drawn. Individuals with schizophrenia have been found typically to show increased volume of the caudate and pallidum compared to healthy controls, so under the assumption that rs10866912 is a risk locus for schizophrenia, it is reasonable to hypothesize that individuals with more risk alleles may be predisposed to possess increased volume in these regions as well. Instead, we find that the number of risk alleles a person possesses actually correlates with decreased gray matter volume in these regions. Regional brain volume is a highly polygenic phenotype, and the association reported here is correspondingly small; rs10866912 is best interpreted as one contributor among many rather than a determinant of pallidal or caudate volume [14]. Its value lies less in the variance explained than in the pathway implicated: the variant was pre-specified based on genome-wide significant discovery and a validated functional effect on NAPRT expression, so even a small association is informative about NAD+ availability. Our findings nonetheless appear to conflict with the literature.

This apparent conflict merits closer examination. The striatal enlargement reported in schizophrenia is substantially confounded by antipsychotic exposure: in antipsychotic-naive patients experiencing a first episode, striatal volumes have been found comparable to those of healthy controls, increasing only after dopamine D2/D3 receptor blockade and in proportion to antipsychotic dose [15]. In a randomized, triple-blind, placebo-controlled trial in antipsychotic-naive first-episode psychosis, right pallidal volume increased over three months in patients randomized to medication and decreased in those randomized to placebo, with no change in healthy controls [16]. Findings in unmedicated samples are not uniform [17], but the direction seen under placebo—pallidal volume declining in the absence of medication—is the direction observed here bilaterally: the left pallidal association reached Bonferroni-corrected significance and the right was nominally significant in the same direction (Table S10). Our sample is drawn from the general population, in which diagnosed psychotic disorder is rare, and excluding the individuals so diagnosed left our associations essentially unchanged. The reduced pallidal and caudate volumes observed in risk-allele carriers therefore need not conflict with schizophrenia neurobiology: the enlargement against which they appear anomalous may be a consequence of treatment rather than a feature of genetic liability.

A developmental mechanism consistent with both the direction and the dose-dependence of these findings is NAD+ insufficiency. The rs10866912 risk allele reduces expression of NAPRT, which catalyses the rate-limiting step of the brain’s principal NAD+ synthesis pathway [2,5]. NAD+ availability constrains mammalian development, and the severity of disruption depends jointly on genetic capacity for NAD+ synthesis and dietary precursor supply [18,19]; severe deficiency is sufficient to produce structural malformation [6]. A milder version of the same constraint in carriers might therefore limit regional growth in proportion to the number of risk alleles carried, which is what we observe. We emphasize that this proposal is speculative, grounded in literature synthesis rather than direct evidence. It does, however, generate testable predictions: that the association should be detectable from early life, should be independent of illness status, and should be modifiable by niacin availability—the last of which connects to the dietary associations reported here.

In addition to the left pallidum and left caudate findings, a number of other brain regions–Intracalcarine cortex (left), anterior cingulate gyrus (right), planum polare (left), caudate (right), pallidum (right), and cerebellum X (right)–surpassed the nominal significance level (*p* < 0.05), suggesting a more diffuse impact of this SNP on brain regions.

When attempting to replicate the dietary findings of our initial study, we were unable to interrogate the additive impact of the risk allele and niacin-poor diet on schizophrenia because schizophrenia cases homozygous for the risk allele in the UK Biobank samples were few in number. However, we investigated the association between the risk allele and diet: while none of the dietary variables assessed met the higher FDR and Bonferroni levels of significance, the variables that did reach nominal significance, increased consumption of wheat and decreased number of years spent vegetarian (grouped), would both result in increased dietary niacin. The converse of a negative correlation between number of risk alleles and years spent as a vegetarian would be a positive correlation between number of risk alleles and years spent as an omnivore. Wheat contains niacin, and meat is exceptionally rich in niacin, so the positive correlations between consumption of these foods and increased number of risk alleles could still be viewed as supporting the hypothesis that, by a yet unknown compensatory mechanism, individuals with a downregulated Preiss–Handler pathway may be more likely to consume niacin-rich food groups in an attempt to upregulate that pathway by providing additional dietary niacin. This finding is consistent with recent research indicating an association between eating wheat products and increased risk of mental disorders [20]. However, the lack of a positive association between egg consumption and number of risk alleles, as seen in the study of 500 individuals with depression, could undermine this, as eggs are also a source of niacin. In all, more research is required to understand any potential relationships between diet, the Preiss–Handler pathway, and risk alleles at rs10866912.

The findings relating to psychological variables and number of risk alleles did not reach any meaningful levels of significance. Nevertheless, having a diagnosis of a psychotic disorder positively correlated with the number of risk alleles at the minimum significance level, which is notable given that this SNP was first identified in a schizophrenia GWAS study of an Indian population.

We acknowledge that despite the overall sample size (*n* = 36,492), this study—like most single-locus association studies—may be underpowered to detect true effects, particularly for the psychiatric and dietary outcomes, where the analytic subsample was substantially smaller (approximately 7000–25,000) than for the neuroimaging analyses. To quantify this, we calculated the minimum effect size detectable with 80% power (Fisher z-transformation method [21]; Figure S13) at each domain’s applicable significance threshold. The minimum detectable correlation was obtained as r = tanh[(z_1−_α/_2_ + z_1−_β)/√(*n*−3)], with z_1−_β = 0.8416 for 80% power and z_1−_α/_2_ evaluated at each domain’s threshold. In the brain-volume domain (*n* = 36,492), effects as small as r = 0.015 (R^2^ ≈ 0.02%) at the nominal threshold, or r = 0.023 (R^2^ ≈ 0.05%) after Bonferroni correction for 139 regions, were detectable. In the psychiatric domain, by contrast, achievable sensitivity ranged from r = 0.018 (R^2^ ≈ 0.03%) at the upper-bound subsample (*n* = 25,000) to r = 0.034 (R^2^ ≈ 0.11%) at the lower-bound subsample (*n* = 7000) at the nominal threshold, widening to r = 0.025–0.047 (R^2^ ≈ 0.06–0.22%) after Bonferroni correction for the 26 variables tested. True associations of a magnitude readily detected in the brain-volume analysis could therefore have been missed in the smaller psychiatric subsamples. We also note that, unlike a conventional candidate-gene screen of numerous, mechanistically uncharacterized variants, rs10866912 was pre-specified on the basis of a genome-wide significant discovery and a validated functional effect on *NAPRT* expression, which narrows—though does not eliminate—the multiple-testing burden typical of the broader candidate-gene literature. The minor allele frequency of rs10866912 also differs substantially between the discovery (Indian, ~72%) and replication (European, ~43%) populations, which may further reduce power to detect an effect of comparable size in this cohort. We therefore interpret the nominal-level psychiatric and dietary associations reported here as hypothesis-generating rather than confirmatory, and highlight the need for replication in an independently ascertained, adequately powered sample.

Two further limitations of the imaging analyses should be noted. First, the adjustment described in Section 2.3.1 is for global gray matter volume rather than for intracranial volume, so variation in cranial capacity is not fully accounted for. Second, imaging center and scanner-related covariates were not available in the analysis dataset and were therefore not included. UK Biobank brain imaging is acquired with identical scanner hardware, software, coils, and protocols at every center, but acquisition site has nonetheless been identified as a potentially important confound in this dataset, as subtle between-site differences may remain [22]; head size and head motion are likewise recognized confounds, and inadequate confound control can induce spurious associations or bias the significance of real ones [22]. Replication with intracranial volume, acquisition site, and head motion included as covariates would strengthen these findings.

## 5. Conclusions

In conclusion, this study advances our understanding of the genetic and environmental contributors to schizophrenia, specifically highlighting the role of the rs10866912 SNP and its association with *NAPRT* expression and the Preiss–Handler pathway. We find a significant association between the risk allele and decreased gray matter volume in the pallidum and caudate, a direction that aligns with observations in unmedicated patients once the effect of antipsychotic treatment on striatal volume is taken into account. Furthermore, the study’s exploration into dietary habits provides intriguing, albeit preliminary, insights into potential compensatory dietary behaviors among individuals with the risk allele, despite not reaching higher levels of statistical significance. The relationship between rs10866912 and the diagnosis of psychotic disorders, while notable, warrants further investigation given the limited sample size of diagnosed individuals.

## Figures and Tables

**Table 1:** Association between rs10866912 risk allele count and gray matter volume in the left pallidum and left caudate, before and after adjustment for total gray matter volume by residualization. β, unstandardized regression coefficient; CI, confidence interval; SE, standard error; FDR, false discovery rate. Em dashes indicate statistics not reported for the unadjusted analyses.

Brain Region	Analysis	β	95% CI	SE	*p*-Value	Significance
Left Pallidum	Original (no TGMV adjustment)	−0.0141	—	—	0.0002	Bonferroni
Left Pallidum	TGMV-adjusted (residualization)	−0.0143	−0.0218 to −0.0069	0.0038	0.00016	Bonferroni
Left Caudate	Original (no TGMV adjustment)	−0.0135	—	—	0.0004	FDR
Left Caudate	TGMV-adjusted (residualization)	−0.0142	−0.0220 to −0.0063	0.0040	0.00038	FDR

## Data Availability

The data analyzed in this study are subject to standard licenses and restrictions. UK Biobank data are available on request to bona fide researchers under an approved application; these data were obtained under UK Biobank Application Number 64593. Requests to access these datasets should be directed to https://www.ukbiobank.ac.uk/ (accessed on 31 March 2023).

## Supplementary Materials

The following supporting information can be downloaded at: https://www.mdpi.com/article/10.3390/genes17101207/s1, Figure S1: Histogram displaying SNP missingness rates in the UKBB subset; Figure S2: Histogram displaying individual missingness rates in the UKBB subset; Figure S3: Distribution of inbreeding coefficients, F, in the UKBB subset; Figure S4: Distribution of inbreeding coefficients in women; Figure S5: Distribution of inbreeding coefficients in samples flagged for discrepant sex; Figure S6: Distribution of minor allele frequency of SNPs on autosomal chromosomes; Figure S7: Distribution of *p*-values from HWE exact tests performed on autosomal SNPs within the UKBB subset; Figure S8: Distribution of heterozygosity rates in the UKBB subset; Figure S9: Distribution of pi-hat values for all pairs of individuals with a pi-hat value greater than or equal to 0.2; Figure S10: The probability of IBD = 0 (Z0) vs the probability of IBD = 1 (Z1) in pairs of individuals with a pi-hat value greater than or equal to 0.2; Figure S11: PCA plots for own samples projected on 1000 Genomes data; Figure S12: Allele frequency plots of UKBB samples vs reference panels; Figure S13: Minimum detectable effect size (80% power) versus sample size for the significance thresholds used in this study; Table S1: Parameters used with Michigan and TOPMed Imputation servers; Table S2: Summary statistics generated by the Imputation Preparation and Checking Tool; Table S3: Summary of problematic sites identified by the checkVCF tool; Table S4: Quality control statistics as reported by the TOPMed Imputation Servers prior to imputation; Table S5: Summary statistics for rs10866912 in each imputed dataset; Table S6: Linear regression coefficients and statistics of statistically significant variables; Table S7: Linear regression statistics for all TOPMed-imputed samples, with domain-specific multiple-testing corrections; Table S8: Linear regression statistics for all TOPMed-imputed samples in individuals with a psychotic disorder; Table S9: Linear regression statistics for all TOPMed-imputed samples in individuals without a psychotic disorder; Table S10: Linear regression statistics for all 139 brain regions using total gray matter volume-adjusted regional volumes. References [20,21,23,24,25,26,27,28,29,30] are cited in the Supplementary Materials.

## Abbreviations

The following abbreviations are used in this manuscript:

BMI	body mass index
FDR	false discovery rate
GWAS	genome-wide association study
IBS	irritable bowel syndrome
MAF	minor allele frequency
MRI	magnetic resonance imaging
NAD+	nicotinamide adenine dinucleotide
NAPRT	nicotinate phosphoribosyltransferase
SNP	single nucleotide polymorphism
TOPMed	Trans-Omics for Precision Medicine
UKBB	UK Biobank
