# Supplementary material for: Association of the Schizophrenia Risk Locus rs10866912 with Cerebral Volumes, Diet, and Psychosis in the UK Biobank
Source: Genes (Basel). 2026 Sep 29;17(10):1207. doi: 10.3390/genes17101207 (PMC13704365; doi:10.3390/genes17101207)
Supplement: Supplementary file 1 [file genes-17-01207-s001.zip › genes-4554510-supplementary.pdf]

Supplementary Information

Table of Contents

Supplementary Methods *2*

GWAS Quality Control 2

Pre-Imputation Quality Control 3

Sample-based QC 3

Pre-Imputation Data Preparation 4

Post-Imputation Quality Control and Variant Isolation 4

Supplementary Results *5*

Sample selection 5

Pre-imputation quality control 5

Assessment of missingness 5

Assessment of sex discrepancies 5

Assessment of minor allele frequency 6

Assessment of deviation from Hardy-Weinberg equilibrium (HWE) 6

Assessment of individual rates of heterozygosity 6

Assessment of relatedness 6

Assessment of population stratification 7

Pre-Imputation Data Preparation 7

Imputation 7

Regression models for individuals with a diagnosis of a psychotic disorder versus individuals without 8

Supplementary Discussion *9*

Limitations and Future Work 12

Supplementary Figures *14*

Figure S1: Histogram displaying SNP missingness rates in the UKBB subset. 14

Figure S2: Histogram displaying individual missingness rates in the UKBB subset. 15

Figure S3: Distribution of inbreeding coefficients, F, in the UKBB subset. 16

Figure S4: Distribution of inbreeding coefficients in women. 17

Figure S5: Distribution of inbreeding coefficients in samples flagged for discrepant sex. 18

Figure S6: Distribution of minor allele frequency of SNPs on autosomal chromosomes. 19

Figure S7: Distribution of p-values from HWE exact tests performed on autosomal SNPs within the UKBB subset. 20

Figure S8: Distribution of heterozygosity rates in the UKBB subset. 21

Figure S9: Distribution of pi-hat values for all pairs of individuals with a pi-hat value greater than or equal to 0.2. 22

Figure S10: The probability of IBD=0 (Z0) vs the probability of IBD=1 (Z1) in pairs of

individuals with a pi-hat value greater than 0.2. ... 23

Figure S11: PCA plots for Own samples projected on 1000Genomes data. ... 24

Figure S12: Allele frequency plots of UKBB samples vs reference panels. ... 25

Figure S13: Minimum detectable effect size (80% power) versus sample size for the significance thresholds used in this study. ... 26

Supplementary Tables ... 26

Table S1: Parameters used with Michigan and TOPMed Imputation servers. ... 26

Table S2: Summary statistics generated by the Imputation Preparation and Checking Tool. ... 26

Table S3: Summary of problematic sites identified by the checkVCF tool. ... 27

Table S4: Quality control statistics as reported by the TOPMed Imputation Servers prior to imputation. ... 27

Table S5: Summary statistics for rs10866912 in each imputed dataset. ... 28

Table S6: Linear regression coefficients and statistics of statistically significant variables. ... 28

Table S7: Linear regression statistics for all TOPMed-imputed samples, with domain-specific multiple-testing corrections. ... 29

Table S8: Linear regression statistics for all TOPMed-imputed samples in individuals with a psychotic disorder. ... 35

Table S9: Linear regression statistics for all TOPMed-imputed samples in individuals without a psychotic disorder. ... 39

Table S10: Linear regression statistics for all 139 brain regions using total gray matter volume-adjusted regional volumes. ... 44

Supplementary Methods

GWAS Quality Control

PLINK-1.9 was used for data manipulation and quality control. The genetic data from the individuals selected for the study were extracted from UKBB, and any individuals who withdrew their data-sharing consent were removed from the sample. Because the UKBB provides individual chromosome files for each subject, the autosomal chromosomes (chromosomes 1-22), sex chromosomes (X and Y chromosomes), including the pseudo-autosomal region of the X chromosome (i.e., PAR or XY), and mitochondrial chromosome files were merged before pre-imputation quality control steps were performed.

Pre-Imputation Quality Control

Pre-imputation quality control was performed using PLINK following the methodology of Marees et al.[1]

Sample-based QC

(i) Missingness. Samples and individual variants with a missingness rate greater than 0.02 were removed; (ii) Sex Discrepancies. Those individuals whose genetic sex did not match their self-reported sex were removed; (iii) Minor Allele Frequency (MAF). SNPs with a MAF less than 0.01 were removed; (iv) Deviation from Hardy–Weinberg equilibrium (HWE). SNPs deviating from HWE with a p-value less than 1e-6 were removed; (v) Individual Rates of Heterozygosity. Regions of high linkage disequilibrium (LD) (the 6:25500000-33500000 HLA region, the 8:8135000-12000000 inversion region, and the 17:40900000-45000000 inversion region) were excluded in order to obtain only independent SNPs. The remaining SNPs were pruned to generate a file containing SNPs not highly correlated. A window size of 50 variant counts was used with a window shift value of 5. The pairwise r^2 threshold was set to 0.02 to prune pairs of variants exceeding this limit. The F-coefficient estimates the distribution of heterozygosity per sample using observed and expected homozygous genotype counts; (vi) Relatedness. Identity-by-descent (IBD) values and proportion IBD (pi-hat) values for each possible pair of individuals within the sample subset were calculated, as was the probability of IBD equaling 0 (Z0), equaling 1 (Z1), and equaling 2 (Z2). A pi-hat value greater than 0.2 indicates cryptic relatedness between the samples. Of the pairs with a pi-hat value greater than 0.2, the call rates of the two individuals were compared to identify and remove the individual with the lower call rate. If two individuals had the same call rate, the first individual in the pair was removed. Pairs with a pi-hat value less than 0.2 were deemed to be unrelated for the purposes of this analysis; (vii) Population Stratification. Two approaches were used: the subjects' self- identified ethnicity, and Principal Component Analysis (PCA) to identify any individuals with a non-European background. The first 10 principal components (PCs) were calculated for individuals in the UKBB subset as well as the 1000 Genomes Project reference samples. The first two PCs in the 1000 Genomes samples were used to determine

the threshold past which UKBB individuals were considered to not be of European ancestry. The centroids of the 1000 Genomes European and American, the nearest neighboring cluster, samples were calculated, and the mid-point of the centroids was used as a PC1 cut-off for UKBB samples. UKBB samples with PC1 greater than the cut-off were removed as outliers.

Pre-Imputation Data Preparation

Genotyping data were appropriately formatted for the imputation servers using the command-line HRC Imputation Preparation and Checking Tool [2]. The HRC reference panel was used, and the program checked for frequency differences, SNP reference/alternate allele mismatches, and issues with strands, alleles, or positions. The merged chromosome files were separated into individual chromosome files for imputation, as only chromosome 8 was needed for this analysis. The binary file set was converted to a VCF file format and a final quality check was performed using the Python program, checkVCF, which identifies inconsistencies with the reference genome.

Imputation

The imputed genotypes were generated using the TOPMed Imputation Servers, which use the genotype imputation software Minimac4.[3-5] On the TOPMed server, the r2 panel was used. Table S1 shows the parameters used for imputation. Imputation was also performed using the Michigan Imputation Server, using the Haplotype Reference Consortium (HRC) r1.1 2016 and 1000 Genomes (1000G) Phase 3v5 panels. The linear regression analysis results described below were obtained using the TOPMed-imputed data, unless otherwise noted.

Post-Imputation Quality Control and Variant Isolation

After imputation, genotypes with low imputation quality (INFO score < 0.8) were removed, and the variant of interest, rs10866912, was isolated in the imputed genotyped data.

The ‘lm’ (linear model) function in R was then used to fit a linear regression model for each

variable, using the variable as a predictor (Xi) and the number of risk alleles at rs10866912 (Yi) as the response, with error ε:

$$Yi = \beta 0 + \beta 1 Xi + \varepsilon$$

Supplementary Results

Sample selection

Of the 502,491 subjects and their associated data in the UKBB, 496,056 participants answered the food group exclusion question (data-field 6144 – "Which of the following do you not eat?"), our first variable of interest. Data were available for the covariates of age, sex, and BMI for 483,705 subjects. A total of 39,690 individuals underwent structural MRI to measure gray matter volumes in various brain regions. A total of 38,467 individuals had answered the diet questionnaire, completed a structural MRI, and reported their age, sex, and BMI. Eight individuals were removed after they withdrew data-sharing consent. Samples were not excluded based on whether they had answered the psychological/psychiatric questions in the UKBB assessment, as it was decided to include these variables later in the study, after sample selection had been performed. Pre-imputation quality control was performed starting with these 38,459 individuals, who collectively had 803,113 SNPs genotyped.

Pre-imputation quality control

Assessment of missingness

Applying a filter to remove SNPs with a missingness rate greater than 2% resulted in the removal of 104,412 SNPs (see Figure S1 for distribution of SNP-level missingness). Applying a filter to remove individuals with a missingness rate greater than 2% resulted in the removal of 244 samples (see Figure S2 for distribution of individual-level missingness).

Assessment of sex discrepancies

Figure S3 shows the expected distribution of inbreeding F-coefficients within the sample. Figure S4 shows the F-coefficients for the females in the group, and Figure S5 shows the

distribution of F-coefficients in a total of 96 samples flagged for discrepant sex. All samples with a sex discrepancy were removed, leaving 38,119 samples for further quality control.

Assessment of minor allele frequency

Removal of SNPs on the sex chromosomes left a total of 681,912 SNPs. MAF was assessed for SNPs on autosomal chromosomes. See Figure S6 for the MAF distribution of autosomal SNPs. SNPs with a MAF less than 1% were removed, resulting in the removal of 99,464 SNPs.

Assessment of deviation from Hardy-Weinberg equilibrium (HWE)

20,435 SNPs with HWE exact test p-values less than 1e-6 were removed, leaving 562,013 SNPs. See Figure S7 showing the distribution of p-values resulting from the HWE exact tests.

Assessment of individual rates of heterozygosity

To avoid calculating inaccurate heterozygosity rates, regions of highly correlated SNPs were pruned, and a total of 300,302 SNPs were removed. Individuals falling outside 3 standard deviations of the heterozygosity rate mean were removed. After 542 samples were removed, 37,577 samples remained for additional quality control. See Figure S8 for the distribution of heterozygosity rates.

Assessment of relatedness

A total of 559 pairs had a pi-hat value of 0.2 or higher, with a mean value of 0.435 and a maximum of 0.999. The individual with a lower genotyping call rate in each cryptically related pair was removed. In some cases, the same individual had the lower call rate in more than one pairing, meaning each pair did not result in the removal of a unique individual. In all, a total of 483 individuals were removed.

See Figure S9 displaying the distribution of pi-hat values greater than or equal to 0.2 in the UKBB subset:

Figure S10 helps visualize the relatedness found within samples by showing the probability of IBD being 0 (Z0) versus the probability of IBD being 1 (Z1) in individual pairs with pi-hat greater than 0.2.

Assessment of population stratification

A total of 602 subjects identified themselves as not being white and were removed from the sample. PCA resulted in the removal of an additional 68 individuals. PC1, PC2, PC3, and PC4 explained 41.6%, 23.0%, 9.90%, and 4.52% of the total variance, respectively. Figure S11A shows PC1 versus PC2 for the 1000 Genomes samples used for comparison. Figure S11B shows the same plot with the UKBB samples and PC1 cut-off overlaid, and Figure S11C shows the UKBB samples overlaid with outliers beyond the cut-off removed. For thoroughness, plots comparing PC2 and PC3 were also generated to ensure removing outliers with a PC1 cut-off was appropriate and effective (see Figures S12A-C).

Pre-Imputation Data Preparation

Several changes were made to the quality-controlled data using the HRC Imputation Preparation and Checking Tool (see Table S2). The dataset was then updated and converted to VCF, in preparation for imputation (See Table S3).

Imputation

Chromosome 8 was submitted for imputation on the TOPMed Imputation Server. See supplementary text and Table S4.

Figures S13A-D show the reference allele frequency plots of the uploaded UKBB samples versus the reference panels. r^2 values measuring the correlation between the allele frequencies of uploaded samples versus allele frequencies of the reference panel for the TOPMed (half 1 and half 2) imputations are as follows: 0.963, 0.963, respectively. There were 16305107 SNPs after imputation. Table S5 shows the quality of imputation for the SNP rs10866912.

The p-value, beta value, and intercept of the linear model with covariates for each brain

region that exceeded the nominal p-value in any of the three imputed datasets can be seen in Table S6.

A table containing the p-values, beta values, r^2 differences (between the model with and without covariates), and intercepts of the linear regression models with covariates for all brain regions analysed, as well as the dietary and psychological variables, can be found in Table S7.

Regression models for individuals with a diagnosis of a psychotic disorder versus individuals without

Observation of the nominally significant, positive correlation between the presence of a psychotic disorder and number of risk alleles prompted the subsequent division of the sample set into individuals with a psychotic disorder diagnosis and those without. Notably, only 80 of the 36,492 individuals had a psychotic disorder diagnosis, so the small sample size and resulting decrease in statistical power must be considered. Individuals with a psychotic disorder were much more likely to possess two risk alleles, A, compared to individuals without a psychotic disorder (33.8% AA vs. 20.25% AA, respectively). Individuals without a psychotic disorder were more likely to have zero risk alleles (30.6% CC) or be heterozygous at this SNP (49.1% CA) than individuals with a psychotic disorder (26.7% CC, 39.6% CA).

Too few individuals with a psychotic disorder reported the number of years they had been vegetarian to obtain results for the vegetarianism variables in this group, though the same significant negative correlations seen previously held in the group without psychotic disorders.

The gray matter volume within one brain region reached nominal significance in the group with psychotic disorders: the right posterior parahippocampal gyrus correlated negatively with the number of risk alleles (i.e., as less gray matter volume is observed, the number of

risk alleles increases). In the control group, we see a small, non-significant negative correlation for this region.

While significant negative correlations between the number of risk alleles and gray matter volume in the left and right pallidum were observed in the control group, this was not seen in the cases group. Similarly, significant negative correlations between the number of risk alleles and gray matter volume in the right and left caudate were seen in the control group, while non-significant positive correlations were seen in the cases group. The significant negative correlation seen with the planum polare in the control group was not significant in the cases group.

Across the different imputations, the gray matter volume in many brain regions showed opposite correlation directions in the cases group compared to the control group. These included the right and left frontal pole, ventral striatum, amygdala, putamen, subcallosal cortex, temporal pole, frontal orbital cortex, left thalamus, left angular gyrus, right occipital pole, and right planum temporale, among others. While not reaching the thresholds for significance, notably, the graygray matter volume in nearly all regions of the cerebellum showed an increase in size with the number of risk alleles in the cases group, while a mix of smaller positive and negative associations were seen in the control group. Table S8 contains the linear regression statistics and coefficients for the psychotic disorder group. Table S9 contains the linear regression statistics and coefficients for the group without psychotic disorders.

Supplementary Discussion

The findings relating to psychological variables and number of risk alleles did not reach any meaningful levels of significance. Having a diagnosis of a psychotic disorder positively correlated with the number of risk alleles at the minimum significance level, which is notable given that this SNP was first identified in a schizophrenia GWAS study of an Indian

population. Only eight individuals in the entire subset analysed had a schizophrenia diagnosis, which is why the decision was made to assess all psychotic disorders, not only schizophrenia.

To further investigate the relationship between regional brain volume differences and this SNP in light of the nominally significant association between number of risk alleles and a psychotic disorder diagnosis, the same linear model regression was performed on individuals with a psychotic disorder and those without. The power of this analysis was considerably limited by only 80 individuals in the subset having a psychotic disorder diagnosis.

Overall, we see many instances of opposite-direction correlations between the psychotic disorder diagnosis cases group and the control group. For example, individuals with a psychotic disorder diagnosis showed a (non-significant) positive correlation with the number of risk alleles for the gray matter volume observed in the left amygdala, while control individuals showed a (non-significant) negative correlation. The findings relating to the cerebellum are of particular note: while controls showed varied strength positive and negative correlations between number of risk alleles and cerebellar regions, cases showed stronger positive correlations in nearly every region of the cerebellum measured, and these findings were consistent throughout the different imputation sets. Once again, this opposes what we may expect given that individuals with schizophrenia, and potentially other psychotic disorders, possess decreased cerebellum gray matter volume.

The converse of a negative correlation between number of risk alleles and years spent as a vegetarian would be a positive correlation between number of risk alleles and years spent as an omnivore. Wheat contains niacin, and meat is exceptionally rich in niacin, so the positive correlations between consumption of these foods and increased number of risk alleles could still be viewed as supporting the hypothesis that, by a yet unknown compensatory

mechanism, individuals with a downregulated Preiss–Handler pathway may be more likely to consume niacin-rich food groups in an attempt to upregulate that pathway by providing additional dietary niacin. However, the lack of a positive association between egg consumption and number of risk alleles, as seen in the study of 500 individuals with depression, could undermine this, as eggs are also a source of niacin. This work is consistent with recent research indicating that eating wheat products may increase the risk of mental disorders.[6]

The right posterior parahippocampal gyrus was negatively correlated with the number of rs10866912 risk alleles at the nominal significance level in the cases group but not the control group. Reduced parahippocampal gray matter has previously been reported, though not consistently,[7] in both chronic[8] and first-episode schizophrenia[9] and in individuals at high risk for psychosis. Moreover, the large multisite ENIGMA meta-analysis reported a small effect size for the parahippocampal gyrus reduction in schizophrenia, while a more recent study using HCP multimodal parcellation reported bilateral thinning of a specific (functional) subregion of the parahippocampal gyrus (PHA3) in FESz[9]. Exploratory correlation analyses suggested that the right PHA3 may be associated with processing speed, working memory, and verbal learning, consistent with known cognitive deficits in schizophrenia.

We can speculate that this finding suggests that an increased number of rs10866912 risk alleles may interfere with normal development of this brain region, via its pivotal role in down-regulating the Preiss–Handler pathway; this would, in turn, reduce NAD concentration, thereby contributing, together with a host of other factors, to the development of a psychotic disorder.

Due to differences in the brain regions that were assessed, direct comparisons cannot be made between the two studies; however, in both studies we see a general decrease in the

gray matter volume of multiple brain regions, notably the caudate and pallidum, as the number of risk alleles at this SNP increases. Performing this same analysis in other populations, namely a population with a lower MAF such as an African or Indian population, would provide interesting data as to whether the gray matter volume in the pallidum and caudate negatively correlates with the number of risk alleles at rs10866912 in non-European individuals. Performing this analysis specifically as a case/control analysis of Indian individuals with and without schizophrenia would be particularly interesting, given the initial identification of this risk locus. This analysis in the European population could be further extended to better identify any links between schizophrenia predispositions and the number of risk alleles if more European schizophrenia cases could be obtained.

Limitations and Future Work

The number of individuals with schizophrenia specifically was extremely limited. With more cases, case/control analyses could have been completed. Future studies should focus on expanding the sample size of individuals with diagnosed psychotic disorders to enhance the statistical power of analyses exploring the relationship between rs10866912 risk alleles and brain morphology in these specific populations. Moreover, longitudinal studies could provide valuable insights into how the rs10866912 risk allele impacts brain development and function over time, potentially clarifying its role in the pathogenesis of schizophrenia. Research into the dietary behaviors of individuals with the risk allele could also elucidate potential compensatory mechanisms aimed at mitigating the downregulation of the Preiss–Handler pathway. Finally, mechanistic studies exploring how the rs10866912 SNP influences NAPRT1 expression and NAD+ biosynthesis could offer critical insights into the biological pathways linking genetic risk factors to schizophrenia's clinical manifestations, ultimately paving the way for targeted therapeutic interventions.

A T1-derived intracranial volume was not available in the analysis dataset, so regional volumes were adjusted for global gray matter volume. Because this measure excludes white matter and cerebrospinal fluid, residual head-size effects cannot be excluded, and

replication using a T1-derived intracranial volume — for example, the UK Biobank volumetric scaling factor — would strengthen these results.

Supplementary Figures

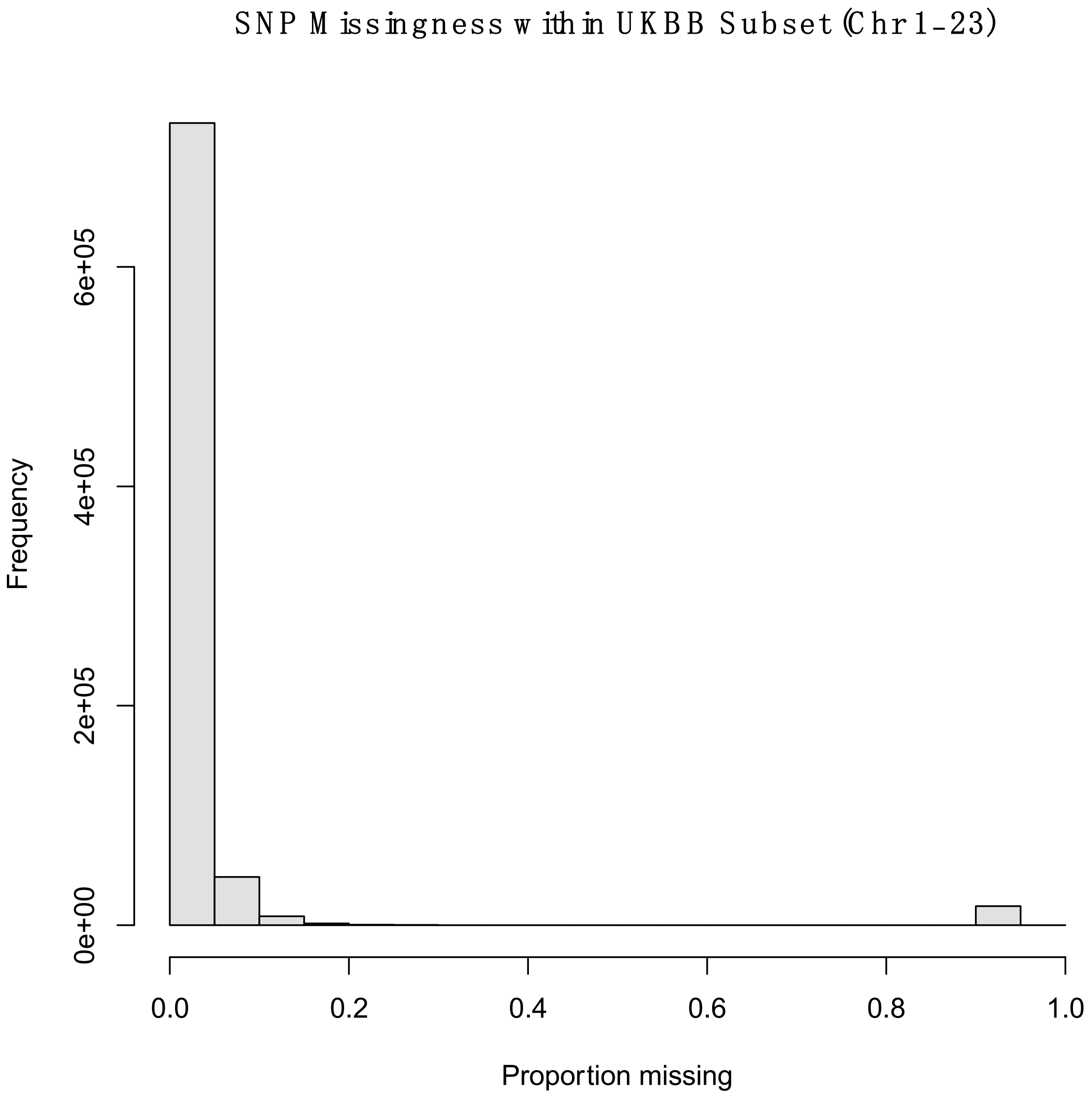

Figure S1: Histogram displaying SNP missingness rates in the UKBB subset.

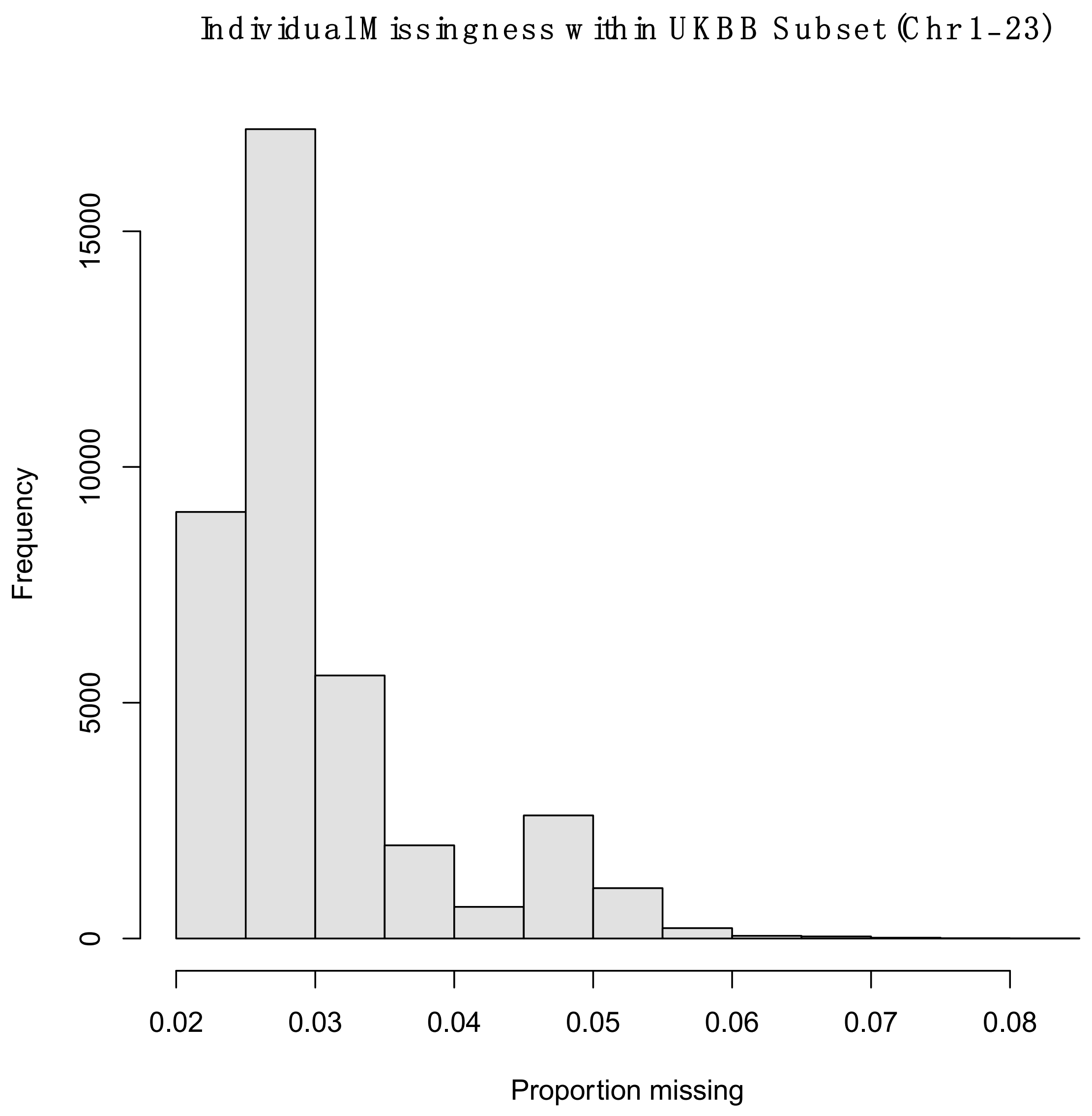

Figure S2: Histogram displaying individual missingness rates in the UKBB subset.

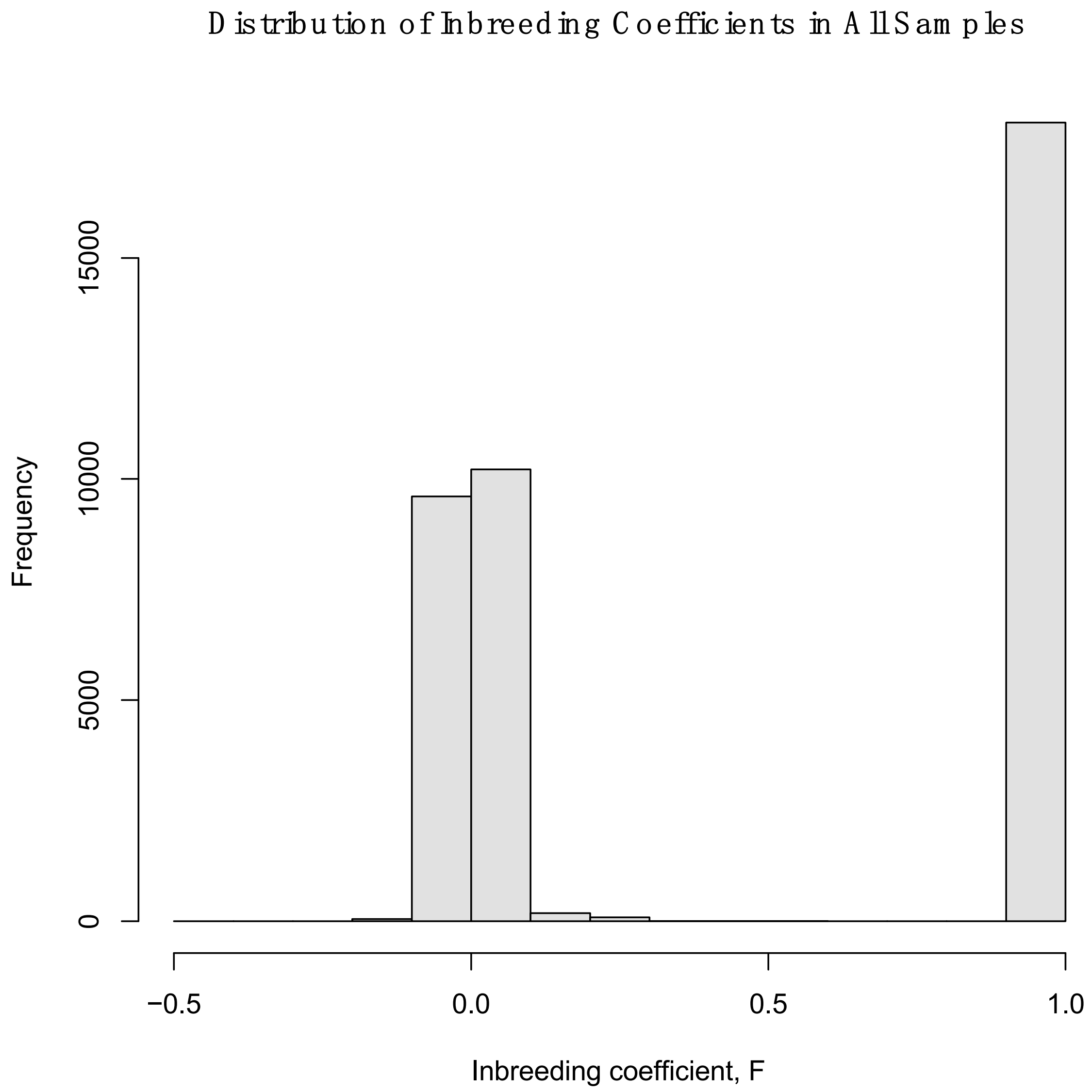

Figure S3: Distribution of inbreeding coefficients, F, in the UKBB subset.

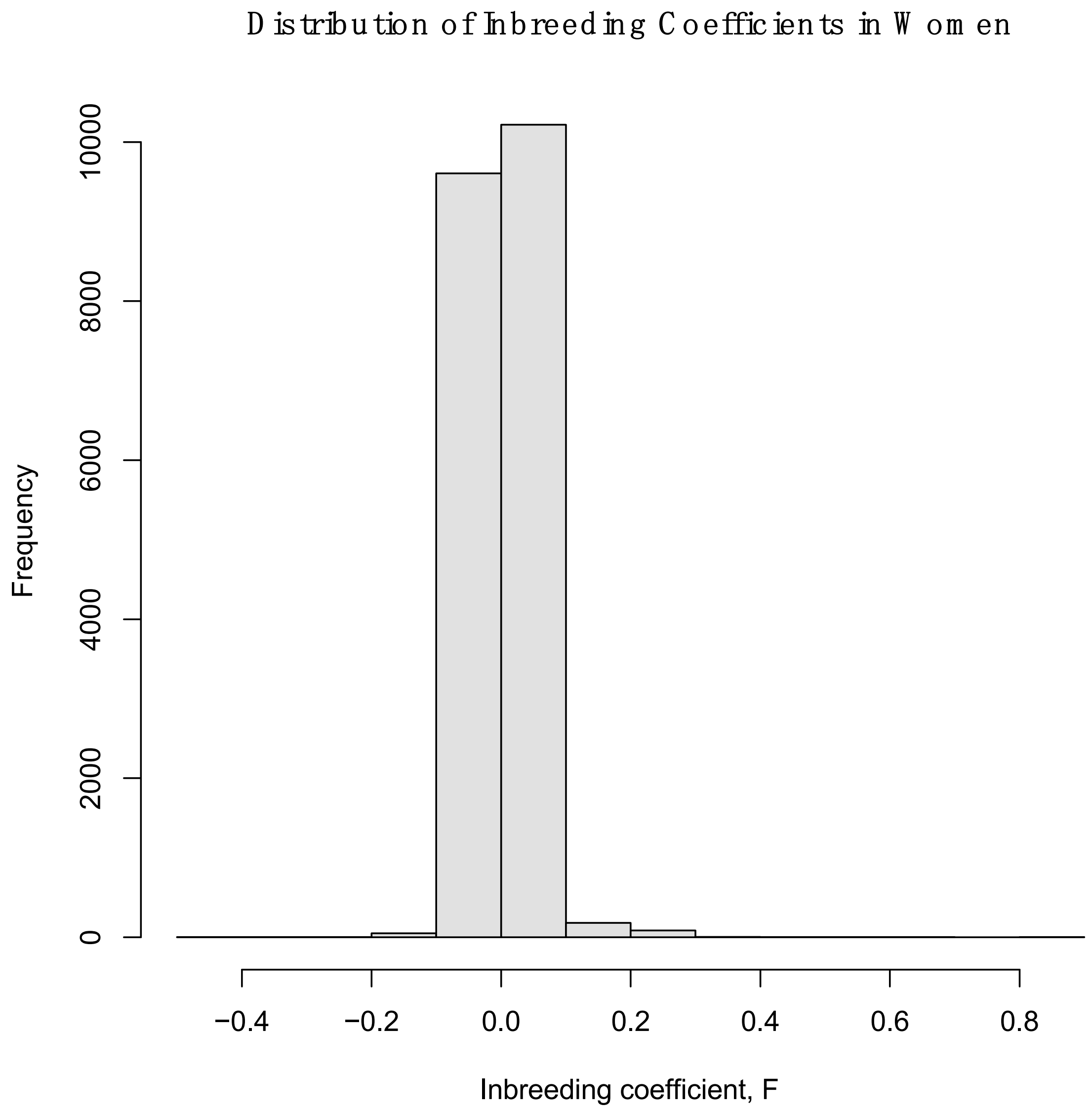

Figure S4: Distribution of inbreeding coefficients in women.

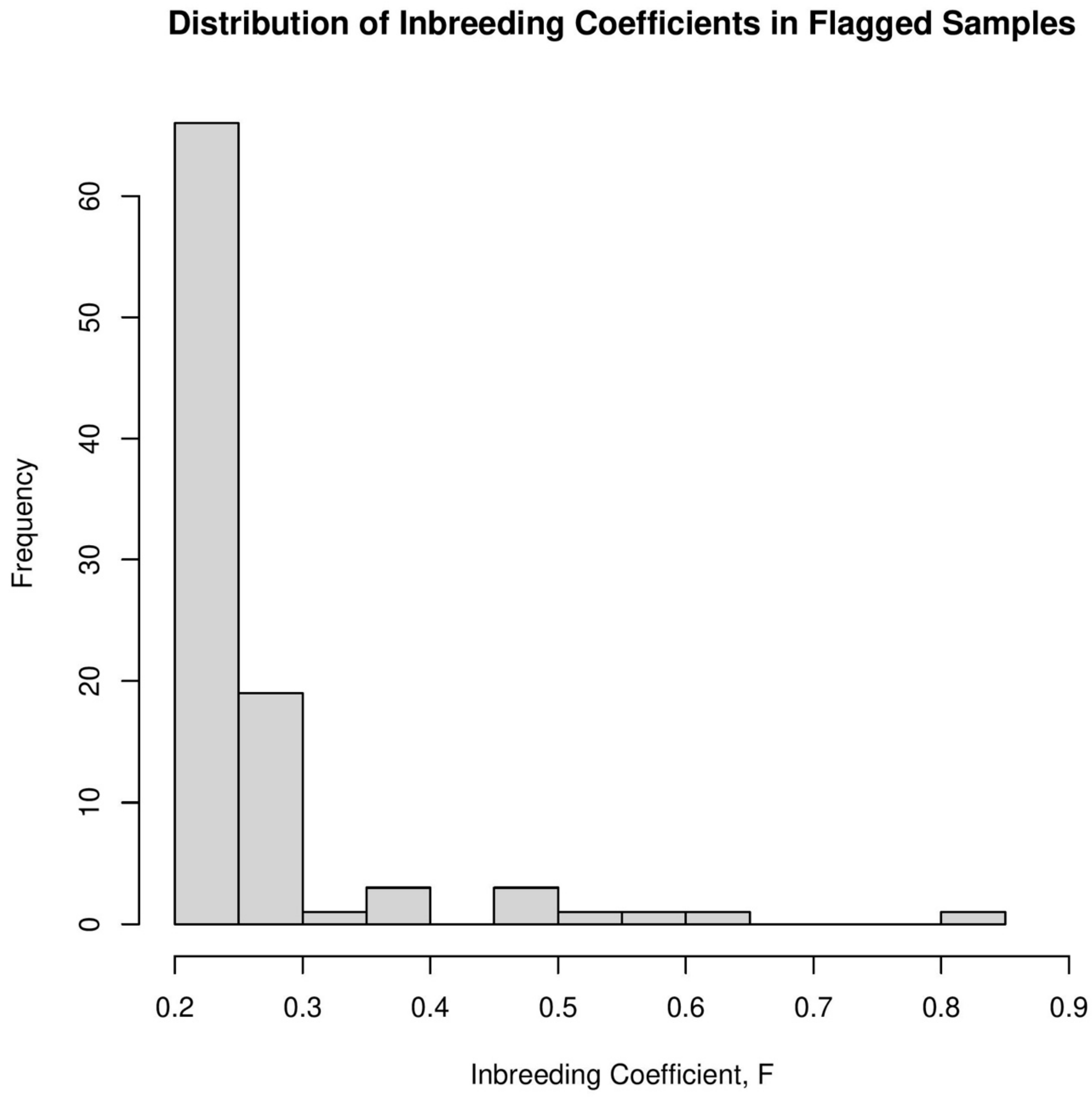

Figure S5: Distribution of inbreeding coefficients in samples flagged for discrepant sex.

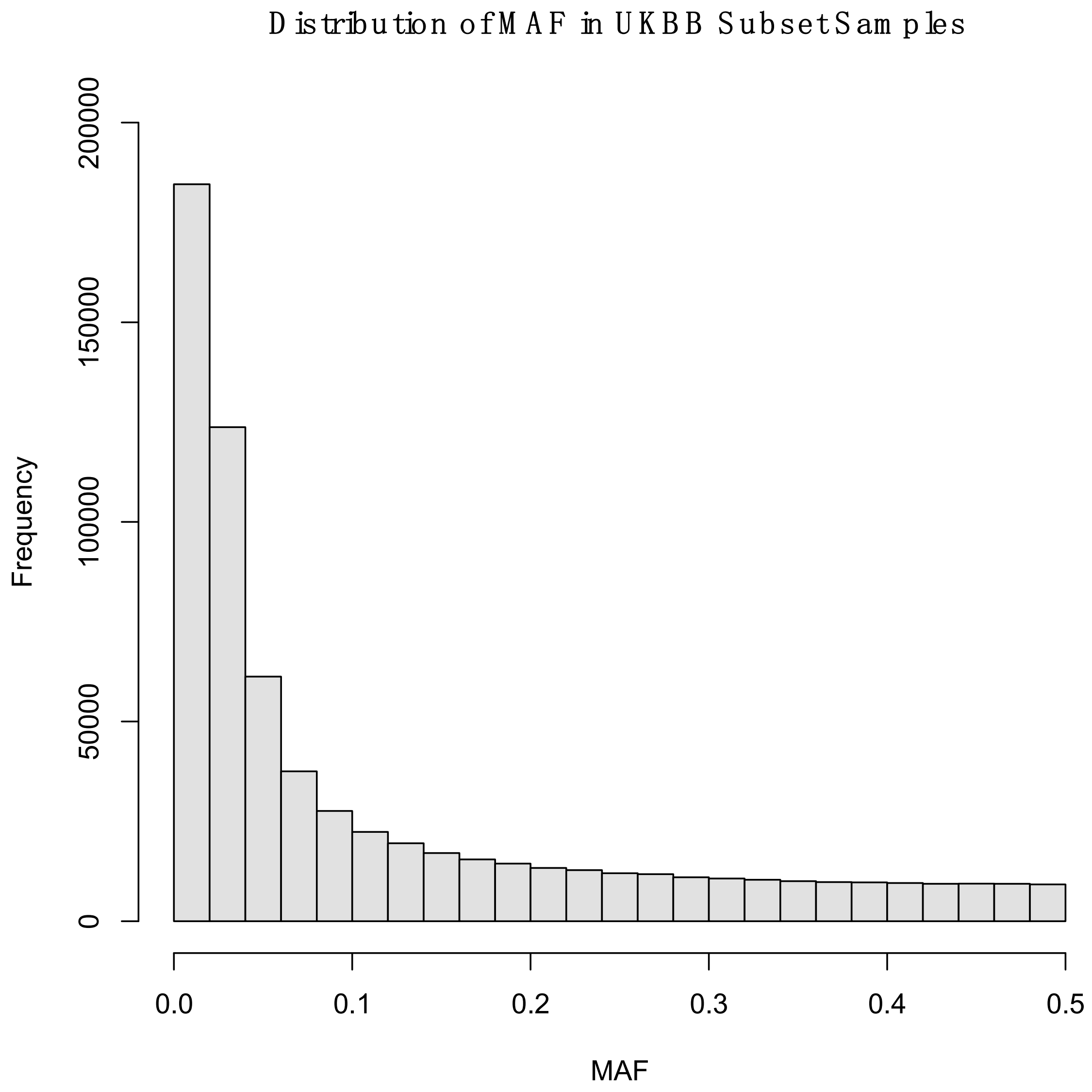

Figure S6: Distribution of minor allele frequency of SNPs on autosomal chromosomes.

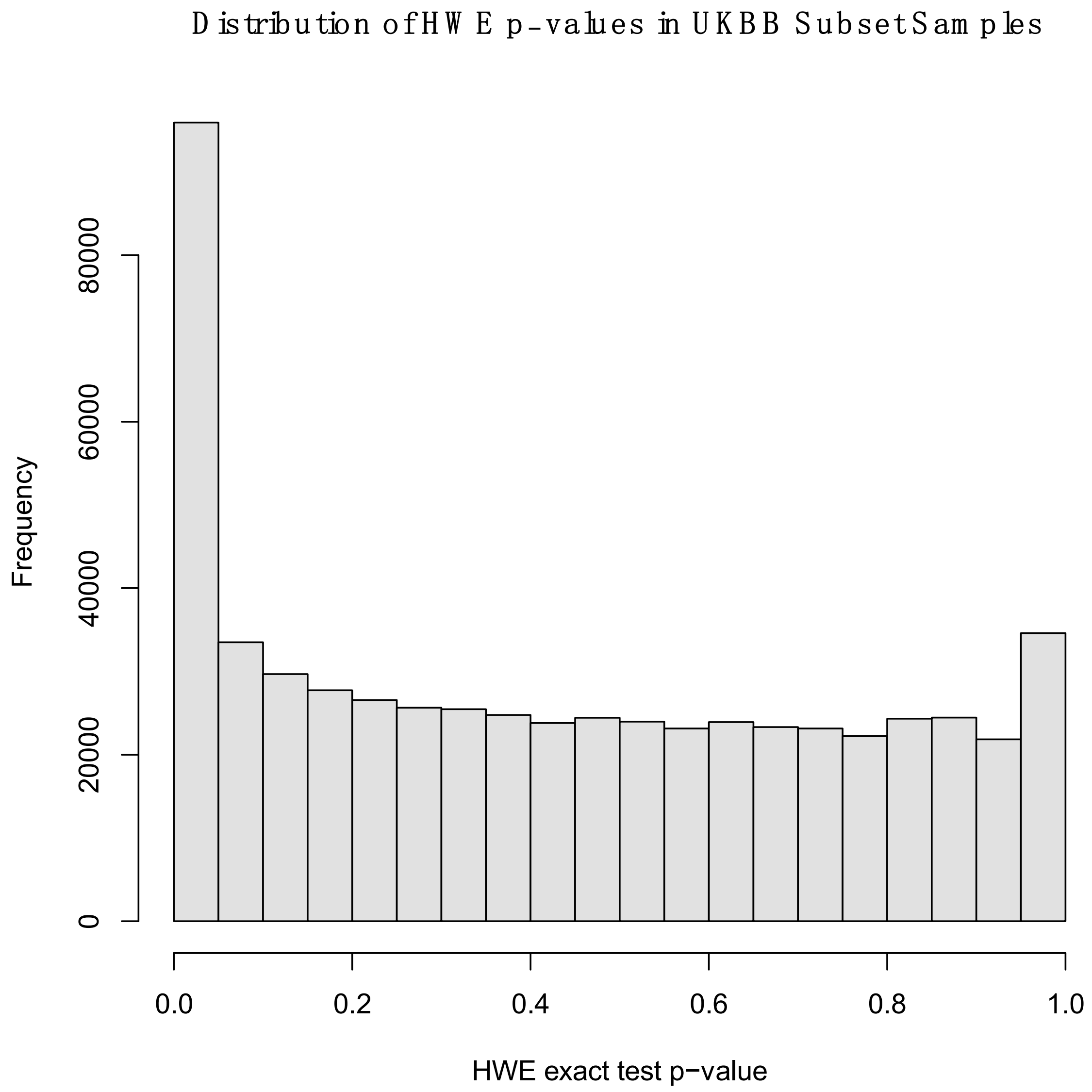

Figure S7: Distribution of p-values from HWE exact tests performed on autosomal SNPs within the UKBB subset.

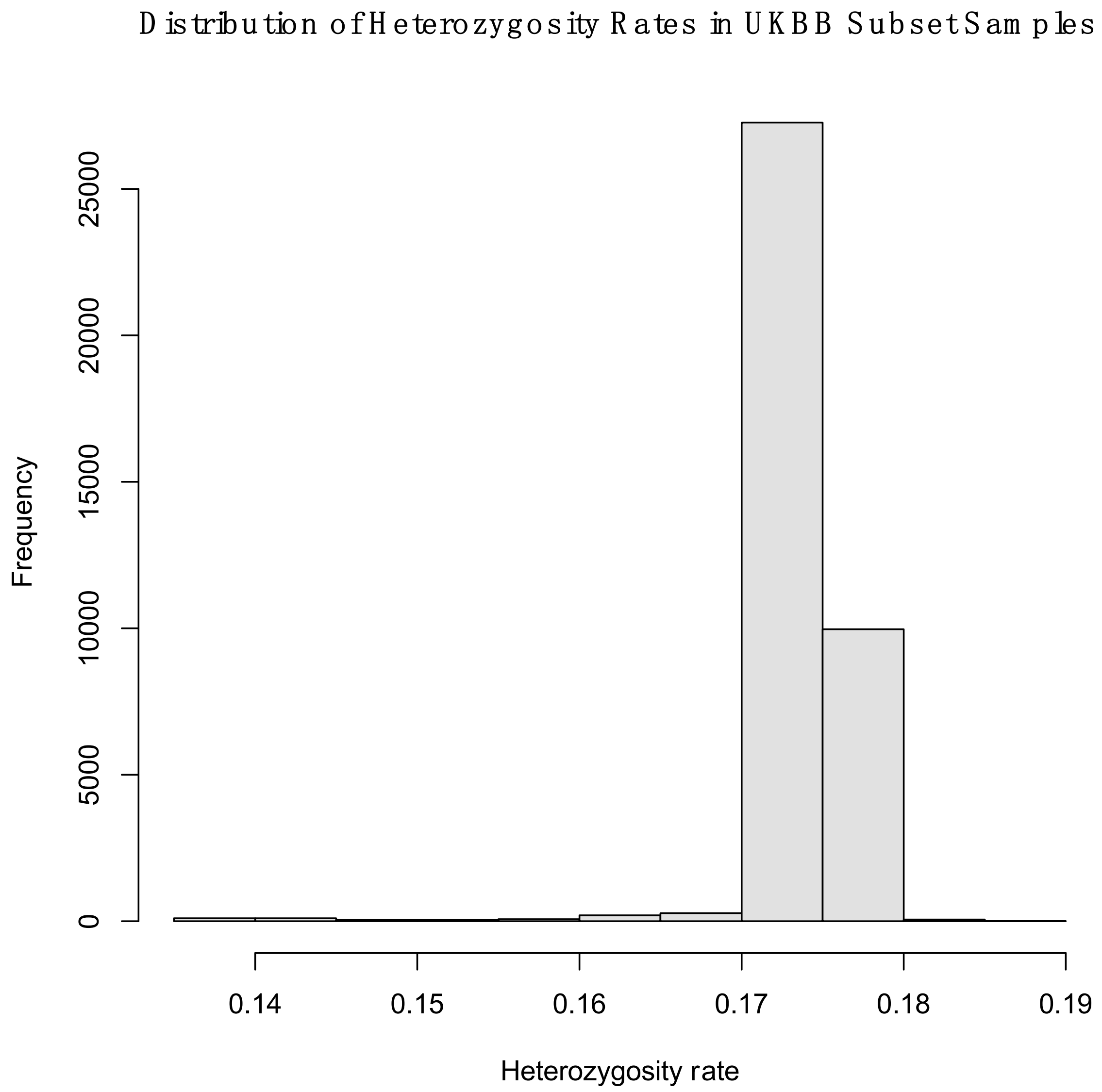

Figure S8: Distribution of heterozygosity rates in the UKBB subset.

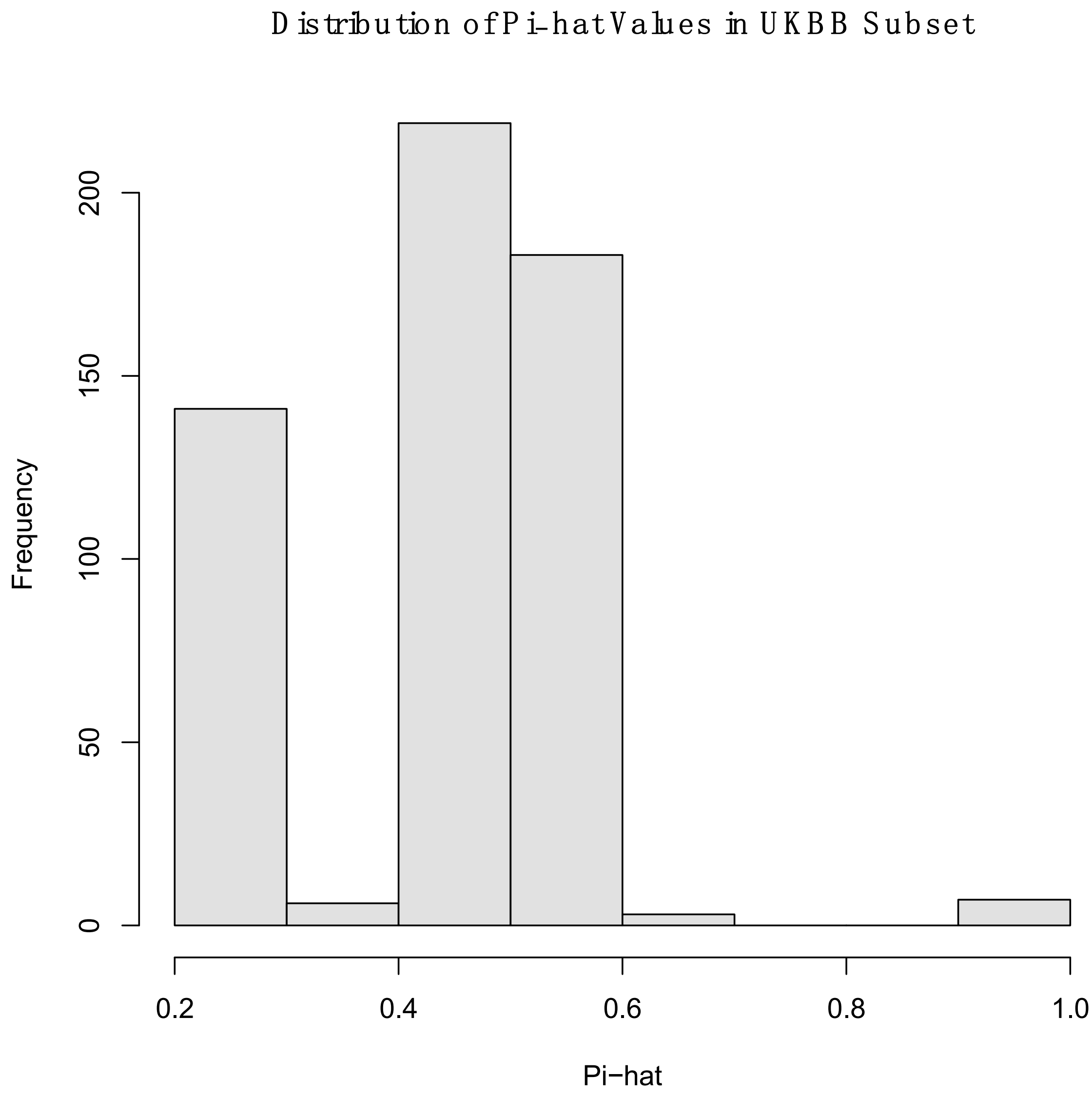

Figure S9: Distribution of pi-hat values for all pairs of individuals with a pi-hat value greater than or equal to 0.2.

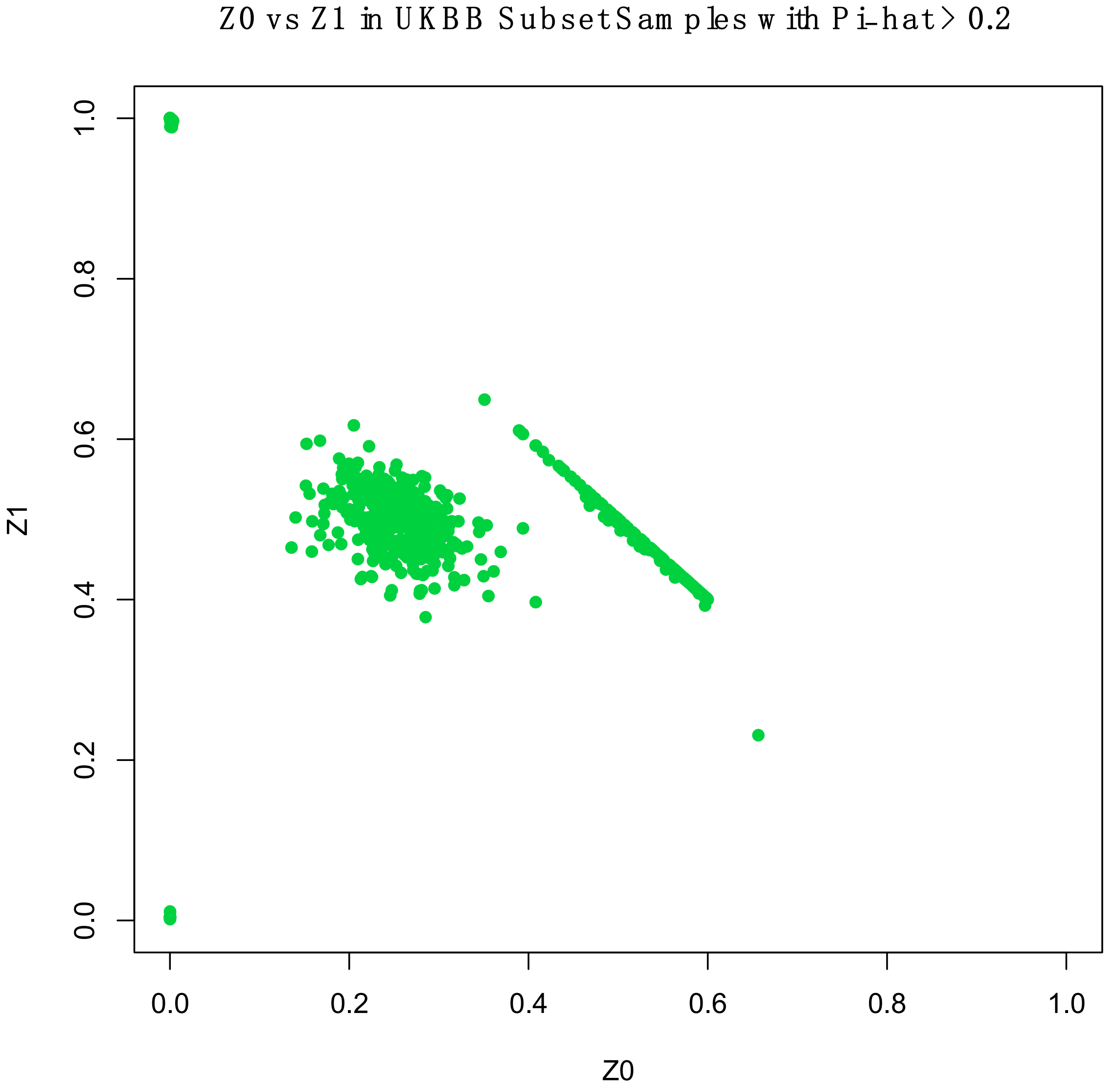

Figure S10: The probability of IBD=0 (Z0) vs the probability of IBD=1 (Z1) in pairs of individuals with a pi-hat value greater than 0.2.

A)

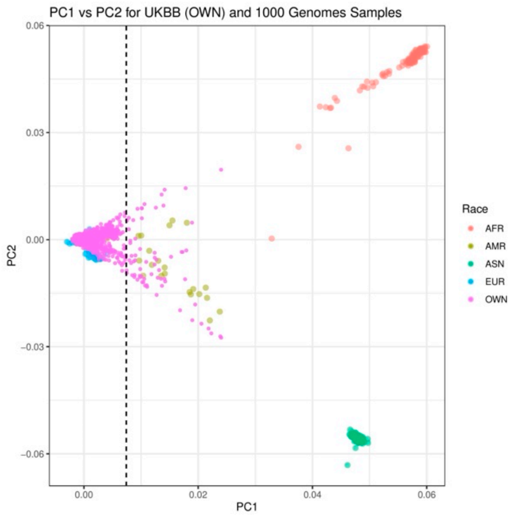

B)

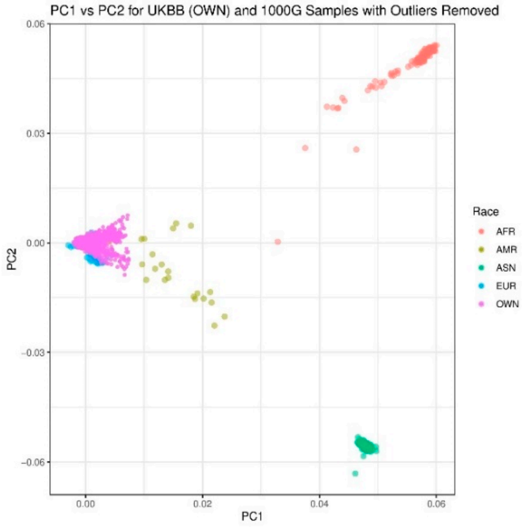

Figure S11: PCA plots for Own samples projected on 1000Genomes data.

A) Plot showing PC1 vs PC2 for race within the 1000 Genomes samples and UKBB samples ("OWN"), as well as the PC1 cut-off for European ancestry based on the centroids of the EUR and AMR clusters. B) Plot showing PC1 vs PC2 for race within the 1000 Genomes samples and UKBB samples with outliers removed.

A) **TOPMed**

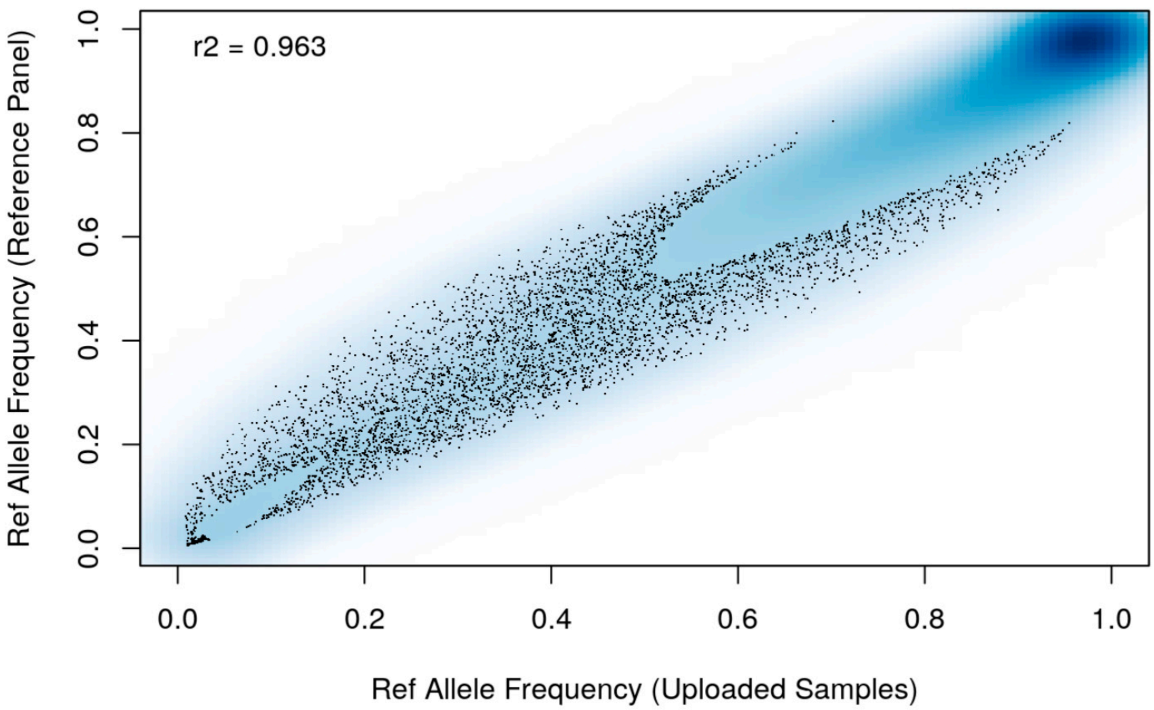

B)

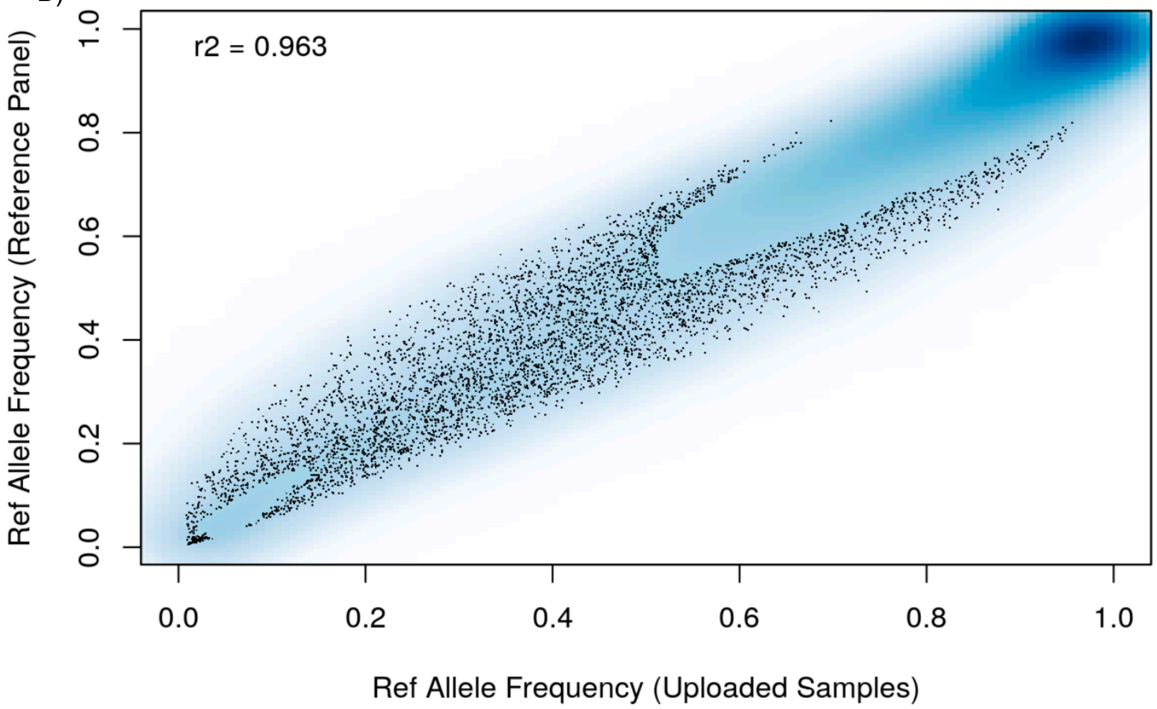

Figure S12: Allele frequency plots of UKBB samples vs reference panels.

Figures S12A and S12B show the allele frequencies using the TOPMed reference panel.

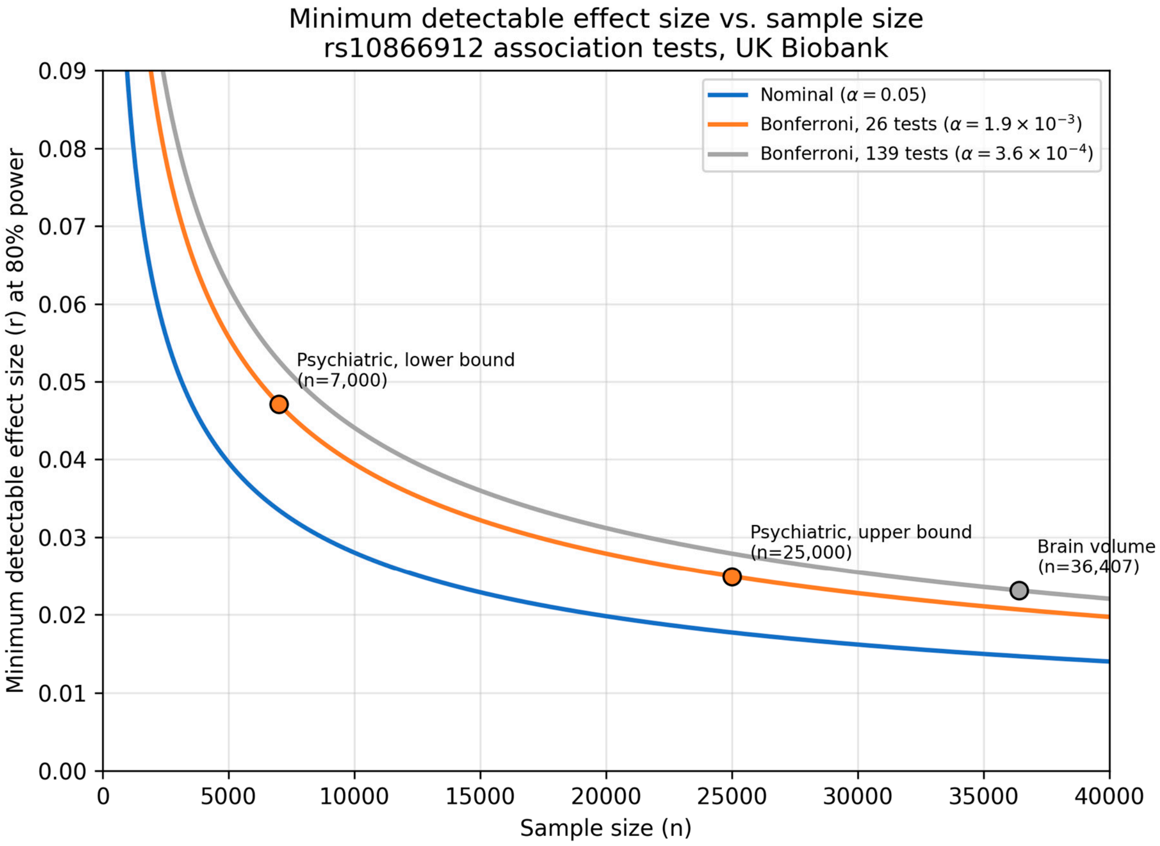

Figure S13: Minimum detectable effect size (80% power) versus sample size for the significance thresholds used in this study.

Minimum detectable correlation (r) at 80% power, plotted against sample size, for the nominal (α=0.05) and Bonferroni-corrected (26 and 139 tests) significance thresholds used in the main text. Points mark the actual sample sizes used for the brain-volume analysis (n=36,492) and the lower and upper bounds of the psychiatric/dietary subsamples (n=7,000 and n=25,000). Minimum detectable effect sizes were calculated using the Fisher z-transformation method[10].

Supplementary Tables

Table S1: Parameters used with Michigan and TOPMed Imputation servers.

Imputation server:	Imputation Settings
Reference panel:	TOPMed r2 (hg38)
Array build:	GRCh37/hg19
Rsq filter:	off
Phasing:	Eagle v2.4 (phased output)
Population:	vs. TOPMed Panel
Mode:	Quality Control & Imputation

Table S2: Summary statistics generated by the Imputation Preparation and Checking Tool.

**Position**	
ID matches HRC	552790

ID doesn't match HRC	4567
Total position matches	557357
**ID Match**	
Different position to HRC	0
No match to HRC	4656
Skipped (X, XY, Y, MT)	0
Total in bim file	562013
Total processed	562013
Indels	0
SNPs not changed	81089
SNPs to change ref alt	472747
Strand ok	553835
Total strand ok	553836
Strand to change	1
Total checked	557357
Total checked strands	553836
Total removed for allele freq diff > 0.2	144
Palindromic SNPs with freq > 0.4	2411
Non-matching alleles	1110
ID and allele mismatching	841
Duplicates removed	0

Table S3: Summary of problematic sites identified by the checkVCF tool.

Duplicated sites	0
Non-SNP sites	0
Inconsistent reference sites	4087
Variant sites with invalid genotypes	0
Alternative allele frequency > 0.5	0
Monomorphic sites	0

Table S4: Quality control statistics as reported by the TOPMed Imputation Servers prior to imputation.

**TOPMed Imputation Server: TOPMed r2**	
**Statistics**	
Alternative allele frequency > 0.5	0
Reference overlap	99.82%
Match	24,499
Allele switch	3912
Strand flip	0
Strand flip and allele switch	0
A/T, C/G genotypes	164
**Filtered sites**	
Invalid alleles	0
Multiallelic sites	0

Duplicated sites	0
Non-SNP sites	0
Monomorphic sites	0
Allele mismatch	14
SNPs call rate < 90%	0
Excluded sites in total	14
Remaining sites in total	28,574

Table S5: Summary statistics for rs10866912 in each imputed dataset.

Imputation Server - Reference Panel	R^2	Alternative allele (C) frequency	MAF (A)
TOPMed – TOPMed (half 1)	0.9788	0.5496	0.4504
TOPMed – TOPMed (half 2)	0.9783	0.5488	0.4512

Table S6: Linear regression coefficients and statistics of statistically significant variables.

	**TOPMed**		
Volume of gray matter within:	Intercept	Beta	P-value
Intracalcarine cortex (left)	0.8894	-0.0078	0.0434
Anterior cingulate gyrus (right)	0.8892	-0.0083	0.0290
Planum polare (left)	0.8891	-0.0116	0.0036
Caudate (left)	0.8889	-0.0135	**0.0004****
Caudate (right)	0.8888	-0.0119	0.0019
Pallidum (left)	0.8905	-0.0141	**0.0002*****
Pallidum (right)	0.8903	-0.0117	0.0023
Cerebellum X (right)	0.8934	0.0095	0.0168

** indicates FDR-corrected significance at $p < 0.05$, *** indicates Bonferroni-corrected significance at $p < 0.05$

Table S7: Linear regression statistics for all TOPMed-imputed samples, with domain-specific multiple-testing corrections.

Risk-allele count (nA) was regressed on each phenotype with age, sex, and BMI as covariates. False discovery rate (Benjamini–Hochberg) and Bonferroni corrections were applied separately within each of the three analytical domains, as described in Methods 2.3.2; the per-domain Bonferroni threshold is given in each section heading. Note that the brain-region coefficients shown here are from the unadjusted analysis; the total gray matter volume-adjusted results for the left pallidum and left caudate are reported in Table 1 of the main text.

Variable	Y-intercept	β coefficient	R^2 difference	p-value	FDR-adjusted p	Bonferroni-adjusted p
**Brain Region Volumes (n = 139 tests; Bonferroni α = 0.05/139 = 3.60E-4)**						
Frontal pole (left)	0.8893	-0.0051	4.03E-05	0.2258	0.8101	1.0000
Frontal pole (right)	0.8905	-0.0018	5.08E-06	0.6670	0.9726	1.0000
Insular cortex (left)	0.8890	-0.0057	5.26E-05	0.1663	0.7410	1.0000
Insular cortex (right)	0.8883	-0.0075	9.18E-05	0.0675	0.7298	1.0000
Superior frontal gyrus (left)	0.8906	-0.0022	9.09E-06	0.5651	0.9726	1.0000
Superior frontal gyrus (right)	0.8905	-0.0026	1.26E-05	0.4984	0.9726	1.0000
Middle frontal gyrus (left)	0.8909	-0.0010	1.99E-06	0.7879	0.9993	1.0000
Middle frontal gyrus (right)	0.8906	-0.0022	8.52E-06	0.5776	0.9726	1.0000
Inferior frontal gyrus: pars triangularis (left)	0.8904	-0.0055	5.72E-05	0.1489	0.7410	1.0000
Inferior frontal gyrus: pars triangularis (right)	0.8907	-0.0032	1.92E-05	0.4035	0.9586	1.0000
Inferior frontal gyrus: pars opercularis (left)	0.8902	-0.0065	7.80E-05	0.0920	0.7298	1.0000
Inferior frontal gyrus: pars opercularis (right)	0.8909	-0.0021	8.43E-06	0.5796	0.9726	1.0000
Precentral gyrus (left)	0.8916	0.0015	3.81E-06	0.7097	0.9726	1.0000
Precentral gyrus (right)	0.8912	0.0002	6.40E-08	0.9615	0.9993	1.0000
Temporal pole (left)	0.8911	0.0000	1.24E-09	0.9946	0.9993	1.0000
Temporal pole (right)	0.8918	0.0018	5.36E-06	0.6585	0.9726	1.0000
Anterior superior temporal gyrus (left)	0.8911	-0.0003	1.46E-07	0.9418	0.9993	1.0000
Anterior superior temporal gyrus (right)	0.8908	-0.0014	3.63E-06	0.7162	0.9726	1.0000
Posterior superior temporal gyrus (left)	0.8905	-0.0029	1.48E-05	0.4631	0.9726	1.0000
Posterior superior temporal gyrus (right)	0.8900	-0.0048	4.00E-05	0.2273	0.8101	1.0000
Anterior middle temporal gyrus (left)	0.8917	0.0017	4.77E-06	0.6768	0.9726	1.0000
Anterior middle temporal gyrus (right)	0.8917	0.0020	7.61E-06	0.5986	0.9726	1.0000
Posterior middle temporal gyrus (left)	0.8907	-0.0016	4.57E-06	0.6834	0.9726	1.0000
Posterior middle temporal gyrus (right)	0.8911	-0.0004	3.20E-07	0.9141	0.9993	1.0000
Middle temporal gyrus, temporo-occipital region (left)	0.8913	0.0008	1.35E-06	0.8244	0.9993	1.0000
Middle temporal gyrus, temporo-occipital region (right)	0.8899	-0.0043	3.21E-05	0.2797	0.8373	1.0000
Anterior inferior temporal gyrus (left)	0.8904	-0.0027	1.33E-05	0.4870	0.9726	1.0000
Anterior inferior temporal gyrus (right)	0.8908	-0.0014	3.67E-06	0.7146	0.9726	1.0000

Variable	Y-intercept	β coefficient	R^2 difference	p-value	FDR-adjusted p	Bonferroni-adjusted p
Posterior inferior temporal gyrus (left)	0.8920	0.0029	1.53E-05	0.4547	0.9726	1.0000
Posterior inferior temporal gyrus (right)	0.8918	0.0024	1.02E-05	0.5421	0.9726	1.0000
Inferior temporal gyrus, temporo-occipital region (left)	0.8927	0.0058	6.03E-05	0.1383	0.7410	1.0000
Inferior temporal gyrus, temporo-occipital region (right)	0.8898	-0.0040	2.75E-05	0.3168	0.8987	1.0000
Postcentral gyrus (left)	0.8904	-0.0054	5.16E-05	0.1706	0.7410	1.0000
Postcentral gyrus (right)	0.8914	0.0019	6.26E-06	0.6331	0.9726	1.0000
Superior parietal lobule (left)	0.8920	0.0059	6.43E-05	0.1260	0.7298	1.0000
Superior parietal lobule (right)	0.8920	0.0056	5.89E-05	0.1432	0.7410	1.0000
Anterior supramarginal gyrus (left)	0.8912	0.0000	2.47E-09	0.9924	0.9993	1.0000
Anterior supramarginal gyrus (right)	0.8913	0.0011	2.41E-06	0.7673	0.9968	1.0000
Posterior supramarginal gyrus (left)	0.8904	-0.0032	1.97E-05	0.3969	0.9586	1.0000
Posterior supramarginal gyrus (right)	0.8909	-0.0015	4.18E-06	0.6966	0.9726	1.0000
Angular gyrus (left)	0.8901	-0.0062	7.43E-05	0.1000	0.7298	1.0000
Angular gyrus (right)	0.8905	-0.0031	1.83E-05	0.4147	0.9586	1.0000
Superior lateral occipital cortex (left)	0.8918	0.0038	2.64E-05	0.3269	0.9088	1.0000
Superior lateral occipital cortex (right)	0.8909	-0.0010	1.75E-06	0.8008	0.9993	1.0000
Inferior lateral occipital cortex (left)	0.8915	0.0015	3.92E-06	0.7054	0.9726	1.0000
Inferior lateral occipital cortex (right)	0.8928	0.0051	4.36E-05	0.2075	0.8081	1.0000
Intracalcarine cortex (left)	0.8894	-0.0078	1.12E-04	0.0434	0.7298	1.0000
Intracalcarine cortex (right)	0.8896	-0.0068	8.61E-05	0.0766	0.7298	1.0000
Frontal medial cortex (left)	0.8917	0.0026	1.19E-05	0.5109	0.9726	1.0000
Frontal medial cortex (right)	0.8916	0.0020	7.46E-06	0.6022	0.9726	1.0000
Juxtapositional lobule cortex (left)	0.8910	-0.0010	1.86E-06	0.7946	0.9993	1.0000
Juxtapositional lobule cortex (right)	0.8903	-0.0047	4.05E-05	0.2248	0.8101	1.0000
Subcallosal cortex (left)	0.8910	-0.0004	2.24E-07	0.9281	0.9993	1.0000
Subcallosal cortex (right)	0.8911	0.0000	2.40E-09	0.9925	0.9993	1.0000
Paracingulate gyrus (left)	0.8900	-0.0044	3.16E-05	0.2831	0.8373	1.0000
Paracingulate gyrus (right)	0.8906	-0.0026	1.12E-05	0.5231	0.9726	1.0000
Anterior cingulate gyrus (left)	0.8899	-0.0054	5.44E-05	0.1593	0.7410	1.0000
Anterior cingulate gyrus (right)	0.8892	-0.0083	1.31E-04	0.0290	0.5759	1.0000
Posterior cingulate gyrus (left)	0.8892	-0.0048	3.61E-05	0.2517	0.8211	1.0000
Posterior cingulate gyrus (right)	0.8883	-0.0072	8.37E-05	0.0808	0.7298	1.0000
Precuneous cortex (left)	0.8923	0.0037	2.29E-05	0.3607	0.9462	1.0000
Precuneous cortex (right)	0.8909	-0.0008	9.46E-07	0.8528	0.9993	1.0000

Variable	Y-intercept	β coefficient	R^2 difference	p-value	FDR-adjusted p	Bonferroni-adjusted p
Cuneal cortex (left)	0.8909	-0.0013	3.33E-06	0.7277	0.9726	1.0000
Cuneal cortex (right)	0.8919	0.0029	1.47E-05	0.4638	0.9726	1.0000
Frontal orbital cortex (left)	0.8904	-0.0021	6.86E-06	0.6173	0.9726	1.0000
Frontal orbital cortex (right)	0.8910	-0.0005	3.33E-07	0.9124	0.9993	1.0000
Anterior parahippocampal gyrus (left)	0.8909	-0.0007	7.38E-07	0.8698	0.9993	1.0000
Anterior parahippocampal gyrus (right)	0.8903	-0.0021	7.22E-06	0.6080	0.9726	1.0000
Posterior parahippocampal gyrus (left)	0.8907	-0.0017	5.05E-06	0.6680	0.9726	1.0000
Posterior parahippocampal gyrus (right)	0.8908	-0.0016	4.74E-06	0.6778	0.9726	1.0000
Lingual gyrus (left)	0.8889	-0.0050	3.84E-05	0.2372	0.8211	1.0000
Lingual gyrus (right)	0.8888	-0.0054	4.54E-05	0.1985	0.8081	1.0000
Anterior temporal fusiform cortex (left)	0.8907	-0.0014	3.46E-06	0.7226	0.9726	1.0000
Anterior temporal fusiform cortex (right)	0.8891	-0.0057	5.49E-05	0.1575	0.7410	1.0000
Posterior temporal fusiform cortex (left)	0.8915	0.0011	2.04E-06	0.7853	0.9993	1.0000
Posterior temporal fusiform cortex (right)	0.8917	0.0016	4.38E-06	0.6897	0.9726	1.0000
Temporal occipital fusiform cortex (left)	0.8893	-0.0065	7.52E-05	0.0979	0.7298	1.0000
Temporal occipital fusiform cortex (right)	0.8900	-0.0036	2.21E-05	0.3695	0.9511	1.0000
Occipital fusiform gyrus (left)	0.8906	-0.0019	6.11E-06	0.6371	0.9726	1.0000
Occipital fusiform gyrus (right)	0.8906	-0.0022	8.53E-06	0.5774	0.9726	1.0000
Frontal operculum cortex (left)	0.8904	-0.0036	2.29E-05	0.3608	0.9462	1.0000
Frontal operculum cortex (right)	0.8897	-0.0068	8.14E-05	0.0852	0.7298	1.0000
Central opercular cortex (left)	0.8897	-0.0051	4.33E-05	0.2093	0.8081	1.0000
Central opercular cortex (right)	0.8901	-0.0043	3.17E-05	0.2830	0.8373	1.0000
Parietal operculum cortex (left)	0.8911	-0.0001	1.26E-08	0.9829	0.9993	1.0000
Parietal operculum cortex (right)	0.8897	-0.0046	3.57E-05	0.2540	0.8211	1.0000
Planum polare (left)	0.8891	-0.0116	2.33E-04	0.0036	0.1001	0.5004
Planum polare (right)	0.8899	-0.0064	6.90E-05	0.1130	0.7298	1.0000
Heschl's gyrus (left)	0.8901	-0.0042	2.90E-05	0.3041	0.8806	1.0000
Heschl's gyrus (right)	0.8902	-0.0039	2.46E-05	0.3439	0.9373	1.0000
Planum temporale (left)	0.8913	0.0005	3.45E-07	0.9108	0.9993	1.0000
Planum temporale (right)	0.8895	-0.0047	3.63E-05	0.2506	0.8211	1.0000
Supracalcarine cortex (left)	0.8896	-0.0065	7.72E-05	0.0936	0.7298	1.0000
Supracalcarine cortex (right)	0.8910	-0.0007	9.81E-07	0.8501	0.9993	1.0000
Occipital pole (left)	0.8910	-0.0005	3.77E-07	0.9067	0.9993	1.0000
Occipital pole (right)	0.8902	-0.0029	1.37E-05	0.4803	0.9726	1.0000

Variable	Y-intercept	β coefficient	R^2 difference	p-value	FDR-adjusted p	Bonferroni-adjusted p
Thalamus (left)	0.8891	-0.0073	9.81E-05	0.0588	0.7298	1.0000
Thalamus (right)	0.8892	-0.0061	6.46E-05	0.1250	0.7298	1.0000
Caudate (left)	0.8889	-0.0135	3.51E-04	0.0004	0.0278	0.0556
Caudate (right)	0.8888	-0.0119	2.65E-04	0.0019	0.0799	0.2641
Putamen (left)	0.8901	-0.0058	6.47E-05	0.1249	0.7298	1.0000
Putamen (right)	0.8906	-0.0031	1.78E-05	0.4205	0.9586	1.0000
Pallidum (left)	0.8905	-0.0141	3.79E-04	0.0002	0.0278	0.0278
Pallidum (right)	0.8903	-0.0117	2.56E-04	0.0023	0.0799	0.3197
Hippocampus (left)	0.8901	-0.0030	1.49E-05	0.4612	0.9726	1.0000
Hippocampus (right)	0.8893	-0.0057	5.39E-05	0.1611	0.7410	1.0000
Amygdala (left)	0.8894	-0.0054	4.57E-05	0.1970	0.8081	1.0000
Amygdala (right)	0.8913	0.0005	3.41E-07	0.9113	0.9993	1.0000
Ventral striatum (left)	0.8904	-0.0033	1.78E-05	0.4207	0.9586	1.0000
Ventral striatum (right)	0.8909	-0.0008	9.44E-07	0.8529	0.9993	1.0000
Brainstem	0.8896	-0.0066	7.08E-05	0.1083	0.7298	1.0000
IIV cerebellum (left)	0.8919	0.0034	2.12E-05	0.3798	0.9586	1.0000
IIV cerebellum (right)	0.8913	0.0008	1.17E-06	0.8366	0.9993	1.0000
V cerebellum (left)	0.8912	0.0002	8.66E-08	0.9552	0.9993	1.0000
V cerebellum (right)	0.8913	0.0006	6.35E-07	0.8792	0.9993	1.0000
VI cerebellum (left)	0.8898	-0.0063	6.76E-05	0.1168	0.7298	1.0000
VI cerebellum (vermis)	0.8915	0.0033	1.99E-05	0.3943	0.9586	1.0000
VI cerebellum (right)	0.8902	-0.0043	3.19E-05	0.2813	0.8373	1.0000
Crus I cerebellum (left)	0.8911	-0.0004	2.78E-07	0.9199	0.9993	1.0000
Crus I cerebellum (vermis)	0.8912	0.0023	1.04E-05	0.5389	0.9726	1.0000
Crus I cerebellum (right)	0.8913	0.0005	4.25E-07	0.9010	0.9993	1.0000
Crus II cerebellum (left)	0.8909	-0.0013	2.98E-06	0.7419	0.9729	1.0000
Crus II cerebellum (vermis)	0.8915	0.0063	7.80E-05	0.0919	0.7298	1.0000
Crus II cerebellum (right)	0.8908	-0.0020	7.26E-06	0.6072	0.9726	1.0000
VIIb cerebellum (left)	0.8908	-0.0024	1.00E-05	0.5454	0.9726	1.0000
VIIb cerebellum (vermis)	0.8912	0.0008	1.16E-06	0.8375	0.9993	1.0000
VIIb cerebellum (right)	0.8911	0.0000	3.81E-09	0.9906	0.9993	1.0000
VIIIa cerebellum (left)	0.8912	0.0002	1.10E-07	0.9496	0.9993	1.0000
VIIIa cerebellum (vermis)	0.8912	0.0002	9.32E-08	0.9536	0.9993	1.0000
VIIIa cerebellum (right)	0.8914	0.0023	9.09E-06	0.5651	0.9726	1.0000

Variable	Y-intercept	β coefficient	R^2 difference	p-value	FDR-adjusted p	Bonferroni-adjusted p
VIIIb cerebellum (left)	0.8908	-0.0024	1.06E-05	0.5339	0.9726	1.0000
VIIIb cerebellum (vermis)	0.8914	0.0020	7.36E-06	0.6047	0.9726	1.0000
VIIIb cerebellum (right)	0.8910	-0.0016	4.53E-06	0.6848	0.9726	1.0000
IX cerebellum (left)	0.8911	-0.0003	2.17E-07	0.9292	0.9993	1.0000
IX cerebellum (vermis)	0.8913	0.0013	3.05E-06	0.7390	0.9729	1.0000
IX cerebellum (right)	0.8911	-0.0015	4.49E-06	0.6860	0.9726	1.0000
X cerebellum (left)	0.8916	0.0016	4.34E-06	0.6910	0.9726	1.0000
X cerebellum (vermis)	0.8912	0.0000	2.08E-11	0.9993	0.9993	1.0000
X cerebellum (right)	0.8934	0.0095	1.57E-04	0.0168	0.3892	1.0000
**Psychological Variables (n = 26 tests; Bonferroni α = 0.05/26 = 1.92E-3)**						
Ever addicted to any substance or behaviour	0.8859	-0.0118	1.50E-05	0.5391	0.9031	1.0000
Lifetime number of depressed periods	0.8861	-0.0035	2.42E-05	0.5634	0.9031	1.0000
Ever taken cannabis	0.8846	-0.0043	3.54E-05	0.3432	0.9031	1.0000
Ever heard an unreal voice	0.8841	0.0428	6.35E-05	0.2050	0.9031	1.0000
Ever believed in an unreal conspiracy against self	0.8844	0.0552	4.51E-05	0.2853	0.9031	1.0000
Ever seen an unreal vision	0.8841	0.0235	3.37E-05	0.3574	0.9031	1.0000
Ever believed in unreal communications or signs	0.8846	-0.0160	3.55E-06	0.7642	0.9031	1.0000
Ever thought that life not worth living	0.8852	-0.0065	8.34E-05	0.1468	0.9031	1.0000
Ever self-harmed	0.8848	-0.0013	1.29E-07	0.9544	0.9544	1.0000
Ever attempted suicide	0.8669	0.0165	1.28E-04	0.7140	0.9031	1.0000
Ever sought or received professional help for mental distress	0.8835	0.0031	4.40E-06	0.7385	0.9031	1.0000
Had/has mental health problems diagnosed by a professional	0.9625	-0.0932	1.24E-04	0.3255	0.9031	1.0000
Ever diagnosed with IBS	0.8897	-0.0022	9.50E-07	0.8709	0.9057	1.0000
Ever been offered or sought treatment for anxiety	0.8908	-0.0079	2.11E-05	0.4379	0.9031	1.0000
Ever been offered or sought treatment for depression	0.8880	0.0018	1.21E-06	0.8528	0.9057	1.0000
Mother had depression	0.8901	0.0180	4.04E-05	0.2285	0.9031	1.0000
Father had depression	0.8921	-0.0120	9.74E-06	0.5559	0.9031	1.0000
Mother had Alzheimer's	0.8907	0.0093	1.14E-05	0.5231	0.9031	1.0000
Father had Alzheimer's	0.8918	-0.0048	1.62E-06	0.8101	0.9057	1.0000
Currently diagnosed with Major Depressive Disorder	0.8999	-0.0237	2.13E-04	0.1662	0.9031	1.0000
Currently diagnosed with bipolar disorder	0.8919	0.0291	1.55E-05	0.7089	0.9031	1.0000
Diagnosis of psychotic disorder	0.8679	0.1649	5.56E-04	0.0380	0.9031	0.9880
Diagnosis of anxiety disorder	0.8744	-0.0123	7.24E-05	0.4539	0.9031	1.0000

Variable	Y-intercept	β coefficient	R^2 difference	p-value	FDR-adjusted p	Bonferroni-adjusted p
Diagnosis of mood disorder	0.8661	0.0059	1.72E-05	0.7153	0.9031	1.0000
Diagnosis of eating disorder	0.8699	-0.0364	2.55E-05	0.6565	0.9031	1.0000
Diagnosis of neurodevelopmental disorder	0.8693	0.1003	3.63E-05	0.5960	0.9031	1.0000
**Dietary Variables (n = 9 tests; Bonferroni α = 0.05/9 = 5.56E-3)**						
Years since meat consumption	0.8683	-0.0320	2.03E-03	0.0549	0.1139	0.4941
Proportion of life spent vegetarian (grouped 0-2)	0.8652	-0.0305	1.90E-03	0.0633	0.1139	0.5697
Years spent vegetarian (grouped 0-2)	0.8672	-0.0349	2.49E-03	0.0333	0.1139	0.2997
Proportion of life spent vegetarian	0.8648	-0.0276	1.53E-03	0.0954	0.1431	0.8586
Age when last ate meat	0.8608	0.0350	2.03E-03	0.0549	0.1139	0.4941
Eats eggs	0.8653	0.0263	2.49E-05	0.3411	0.4386	1.0000
Eats dairy	0.8893	0.0019	7.12E-08	0.9594	0.9594	1.0000
Eats wheat	0.8216	0.0708	1.28E-04	0.0311	0.1139	0.2799
Eats sugar	0.8925	-0.0015	5.40E-07	0.8885	0.9594	1.0000

Table S8: Linear regression statistics for all TOPMed-imputed samples in individuals with a psychotic disorder.

TOPMed	Y-intercept	Beta coefficient	R^2 difference	P-value
Brain Region Volumes<br>Volume of gray matter within:				
Frontal pole (left)	1.0705	1.39E-01	0.0228	0.1828
Frontal pole (right)	1.0394	2.64E-02	0.0007	0.814
Insular cortex (left)	1.0142	-2.92E-02	0.0009	0.7941
Insular cortex (right)	0.9872	-7.49E-02	0.0051	0.5289
Superior frontal gyrus (left)	1.0504	6.31E-02	0.0051	0.5311
Superior frontal gyrus (right)	1.0311	8.17E-03	0.0001	0.9333
Middle frontal gyrus (left)	0.9988	-8.44E-02	0.0093	0.3956
Middle frontal gyrus (right)	1.0210	-4.82E-02	0.0033	0.615
Inferior frontal gyrus: pars triangularis (left)	1.0076	-8.02E-02	0.0087	0.4132
Inferior frontal gyrus: pars triangularis (right)	1.0404	6.41E-02	0.0057	0.5057
Inferior frontal gyrus: pars opercularis (left)	1.0304	9.57E-03	0.0001	0.9231
Inferior frontal gyrus: pars opercularis (right)	1.0458	6.52E-02	0.0062	0.488
Precentral gyrus (left)	1.0236	-1.89E-02	0.0005	0.8469
Precentral gyrus (right)	1.0294	8.35E-03	0.0001	0.9295
Temporal pole (left)	1.0891	1.47E-01	0.0251	0.1618
Temporal pole (right)	0.9971	-8.87E-02	0.0101	0.3768
Anterior superior temporal gyrus (left)	1.0034	-7.93E-02	0.0080	0.4302
Anterior superior temporal gyrus (right)	1.0114	-7.41E-02	0.0075	0.4462
Posterior superior temporal gyrus (left)	1.0620	9.22E-02	0.0109	0.3589
Posterior superior temporal gyrus (right)	1.0446	7.71E-02	0.0081	0.4281
Anterior middle temporal gyrus (left)	1.0290	2.96E-03	0.0000	0.9755
Anterior middle temporal gyrus (right)	1.0196	-6.66E-02	0.0065	0.4778
Posterior middle temporal gyrus (left)	1.0421	7.21E-02	0.0079	0.4355
Posterior middle temporal gyrus (right)	1.0345	1.09E-01	0.0182	0.2343
Middle temporal gyrus, temporo-occipital region (left)	1.0312	3.61E-02	0.0019	0.6989
Middle temporal gyrus, temporo-occipital region (right)	1.0131	-4.60E-02	0.0028	0.6409
Anterior inferior temporal gyrus (left)	1.0273	-1.43E-02	0.0003	0.874
Anterior inferior temporal gyrus (right)	1.0272	-1.38E-02	0.0003	0.8784
Posterior inferior temporal gyrus (left)	1.0170	-6.36E-02	0.0064	0.4827
Posterior inferior temporal gyrus (right)	1.0441	1.14E-01	0.0201	0.2112
Inferior temporal gyrus, temporo-occipital region (left)	1.0620	1.08E-01	0.0160	0.2657
Inferior temporal gyrus, temporo-occipital region (right)	1.0677	9.70E-02	0.0114	0.3483
Postcentral gyrus (left)	1.0217	-3.13E-02	0.0012	0.7567
Postcentral gyrus (right)	1.0288	5.12E-03	0.0000	0.9564
Superior parietal lobule (left)	1.0231	-4.59E-02	0.0033	0.6137

Superior parietal lobule (right)	1.0298	1.29E-02	0.0003	0.8861
Anterior supramarginal gyrus (left)	1.0373	1.54E-01	0.0381	0.0837
Anterior supramarginal gyrus (right)	1.0330	2.95E-02	0.0013	0.7562
Posterior supramarginal gyrus (left)	1.0343	9.78E-02	0.0150	0.28
Posterior supramarginal gyrus (right)	1.0441	5.84E-02	0.0046	0.5516
Angular gyrus (left)	1.0390	9.82E-02	0.0146	0.2865
Angular gyrus (right)	1.0158	-4.69E-02	0.0031	0.6248
Superior lateral occipital cortex (left)	1.0306	9.88E-03	0.0001	0.9191
Superior lateral occipital cortex (right)	1.0270	-5.05E-03	0.0000	0.9592
Inferior lateral occipital cortex (left)	1.0614	8.87E-02	0.0102	0.375
Inferior lateral occipital cortex (right)	1.0471	8.46E-02	0.0106	0.3641
Intracalcarine cortex (left)	1.0344	2.73E-02	0.0011	0.7701
Intracalcarine cortex (right)	1.0222	-3.36E-02	0.0018	0.712
Frontal medial cortex (left)	1.0354	3.55E-02	0.0019	0.7019
Frontal medial cortex (right)	1.0253	-4.16E-02	0.0027	0.6482
Juxtapositional lobule cortex (left)	1.0326	2.75E-02	0.0011	0.776
Juxtapositional lobule cortex (right)	1.0457	8.13E-02	0.0087	0.4114
Subcallosal cortex (left)	1.0436	4.85E-02	0.0029	0.6345
Subcallosal cortex (right)	1.0748	1.22E-01	0.0189	0.2256
Paracingulate gyrus (left)	1.0307	9.16E-03	0.0001	0.93
Paracingulate gyrus (right)	1.0297	4.83E-03	0.0000	0.9639
Anterior cingulate gyrus (left)	1.0228	-2.72E-02	0.0012	0.7657
Anterior cingulate gyrus (right)	1.0295	7.00E-03	0.0001	0.9403
Posterior cingulate gyrus (left)	1.0642	1.14E-01	0.0154	0.2736
Posterior cingulate gyrus (right)	1.0648	1.35E-01	0.0253	0.1597
Precuneous cortex (left)	1.0453	7.06E-02	0.0064	0.4803
Precuneous cortex (right)	1.0142	-5.53E-02	0.0039	0.581
Cuneal cortex (left)	1.0308	9.00E-03	0.0001	0.9276
Cuneal cortex (right)	1.0068	-6.10E-02	0.0042	0.5702
Frontal orbital cortex (left)	1.0684	1.04E-01	0.0114	0.3481
Frontal orbital cortex (right)	1.0584	8.48E-02	0.0088	0.409
Anterior parahippocampal gyrus (left)	1.1094	1.86E-01	0.0378	0.085
Anterior parahippocampal gyrus (right)	1.0184	-2.32E-02	0.0007	0.8214
Posterior parahippocampal gyrus (left)	0.9731	-1.89E-01	0.0385	0.0821
Posterior parahippocampal gyrus (right)	0.9790	-2.02E-01	0.0525	0.0415
Lingual gyrus (left)	1.0637	1.43E-01	0.0304	0.123
Lingual gyrus (right)	1.1175	1.97E-01	0.0399	0.0764
Anterior temporal fusiform cortex (left)	1.0482	9.44E-02	0.0136	0.305
Anterior temporal fusiform cortex (right)	1.0197	-2.89E-02	0.0012	0.7651
Posterior temporal fusiform cortex (left)	1.0215	-2.12E-02	0.0005	0.8437
Posterior temporal fusiform cortex (right)	1.0063	-7.11E-02	0.0058	0.5023
Temporal occipital fusiform cortex (left)	1.0316	1.02E-02	0.0001	0.9163
Temporal occipital fusiform cortex (right)	1.0416	3.73E-02	0.0017	0.7193
Occipital fusiform gyrus (left)	1.1001	1.86E-01	0.0438	0.0632
Occipital fusiform gyrus (right)	1.0406	6.98E-02	0.0073	0.4513
Frontal operculum cortex (left)	1.0320	1.76E-02	0.0004	0.8576

Frontal operculum cortex (right)	1.0355	3.01E-02	0.0011	0.7662
Central opercular cortex (left)	0.9987	-1.11E-01	0.0168	0.2539
Central opercular cortex (right)	1.0324	1.83E-02	0.0005	0.8459
Parietal operculum cortex (left)	1.0132	-3.57E-02	0.0013	0.7491
Parietal operculum cortex (right)	0.9897	-1.08E-01	0.0145	0.2884
Planum polare (left)	1.0160	-4.38E-02	0.0025	0.659
Planum polare (right)	1.0386	3.83E-02	0.0019	0.7049
Heschl's gyrus (left)	1.0118	-5.62E-02	0.0040	0.5774
Heschl's gyrus (right)	1.0145	-5.10E-02	0.0036	0.6005
Planum temporale (left)	1.0353	1.54E-02	0.0003	0.8892
Planum temporale (right)	1.0682	1.09E-01	0.0140	0.2972
Supracalcarine cortex (left)	1.0417	6.14E-02	0.0052	0.5258
Supracalcarine cortex (right)	1.0335	1.93E-02	0.0005	0.8471
Occipital pole (left)	1.0052	-8.70E-02	0.0107	0.3633
Occipital pole (right)	1.0506	5.56E-02	0.0039	0.5815
Thalamus (left)	1.0324	1.37E-02	0.0003	0.8852
Thalamus (right)	1.0206	-2.33E-02	0.0007	0.8101
Caudate (left)	1.0371	8.29E-02	0.0110	0.3557
Caudate (right)	1.0400	6.73E-02	0.0069	0.4638
Putamen (left)	1.0489	6.32E-02	0.0056	0.5095
Putamen (right)	1.0350	2.74E-02	0.0011	0.7686
Pallidum (left)	1.0243	-6.05E-02	0.0058	0.5044
Pallidum (right)	1.0267	-8.36E-03	0.0001	0.9287
Hippocampus (left)	1.0150	-5.77E-02	0.0044	0.5585
Hippocampus (right)	0.9995	-1.21E-01	0.0204	0.208
Amygdala (left)	1.0736	1.41E-01	0.0236	0.175
Amygdala (right)	1.0164	-3.94E-02	0.0019	0.6984
Ventral striatum (left)	1.0503	1.40E-01	0.0228	0.1829
Ventral striatum (right)	1.0661	1.34E-01	0.0210	0.2014
Brainstem	0.9908	-1.26E-01	0.0190	0.2237
IIV cerebellum (left)	1.0246	-2.56E-02	0.0010	0.7803
IIV cerebellum (right)	1.0284	1.11E-03	0.0000	0.9904
V cerebellum (left)	1.0186	-6.17E-02	0.0053	0.5226
V cerebellum (right)	1.0195	-3.79E-02	0.0019	0.6999
VI cerebellum (left)	1.0253	-1.47E-02	0.0003	0.8883
VI cerebellum (vermis)	1.0304	9.23E-02	0.0133	0.3097
VI cerebellum (right)	1.0185	-4.21E-02	0.0024	0.6702
Crus I cerebellum (left)	1.0545	1.44E-01	0.0275	0.1425
Crus I cerebellum (vermis)	1.0278	-3.58E-02	0.0021	0.6903
Crus I cerebellum (right)	1.0533	1.22E-01	0.0169	0.2514
Crus II cerebellum (left)	1.0462	9.14E-02	0.0102	0.3745
Crus II cerebellum (vermis)	1.0360	1.06E-01	0.0175	0.2441
Crus II cerebellum (right)	1.0375	5.87E-02	0.0042	0.5704
VIIb cerebellum (left)	1.0433	1.20E-01	0.0187	0.228
VIIb cerebellum (vermis)	1.0264	9.90E-02	0.0146	0.2866
VIIb cerebellum (right)	1.0438	9.39E-02	0.0118	0.3381

VIIIa cerebellum (left)	1.0340	7.75E-02	0.0074	0.4502
VIIIa cerebellum (vermis)	1.0254	7.59E-02	0.0080	0.4315
VIIIa cerebellum (right)	1.0300	3.22E-02	0.0014	0.7396
VIIIb cerebellum (left)	1.0388	6.72E-02	0.0060	0.4948
VIIIb cerebellum (vermis)	1.0323	8.53E-02	0.0106	0.3654
VIIIb cerebellum (right)	1.0308	5.28E-02	0.0038	0.586
IX cerebellum (left)	1.0295	2.42E-02	0.0008	0.8015
IX cerebellum (vermis)	1.0285	2.07E-03	0.0000	0.9833
IX cerebellum (right)	1.0299	4.57E-02	0.0030	0.6292
X cerebellum (left)	0.9975	-1.11E-01	0.0165	0.2583
X cerebellum (vermis)	1.0288	1.11E-02	0.0002	0.9023
X cerebellum (right)	0.9898	-1.81E-01	0.0421	0.0686
Psychological Variables				
Ever addicted to any substance or behaviour	1.1106	-2.77E-01	0.0195	0.2263
Lifetime number of depressed periods	0.9984	7.87E-02	0.0098	0.4081
Ever taken cannabis	1.0156	-1.02E-01	0.0152	0.2779
Ever heard an unreal voice	1.0725	-9.69E-02	0.0034	0.6181
Ever believed in an unreal conspiracy against self	1.0799	-1.50E-01	0.0078	0.437
Ever seen an unreal vision	1.1753	-3.47E-01	0.0364	0.0985
Ever believed in unreal communications or signs	1.0180	3.95E-02	0.0006	0.8366
Ever thought that life not worth living	1.0368	-5.18E-02	0.0040	0.5799
Ever self-harmed	1.0246	8.68E-03	0.0000	0.9658
Ever attempted suicide	1.7279	-7.82E-01	0.1949	0.0257
Ever sought or received professional help for mental distress	1.0732	-4.33E-02	0.0000	0.9572
Ever diagnosed with IBS	0.9370	2.29E-01	0.0080	0.477
Ever been offered or sought treatment for anxiety	0.9060	1.21E-01	0.0048	0.5758
Ever been offered or sought treatment for depression	1.1942	-2.37E-01	0.0146	0.3202
Mother had depression	1.0947	-2.47E-01	0.0185	0.237
Father had depression	1.0325	-3.95E-02	0.0002	0.8959
Mother had Alzheimer's	1.0391	-1.34E-01	0.0009	0.7947
Father had Alzheimer's	1.0371	-1.15E-01	0.0013	0.7584
Currently diagnosed with Major Depressive Disorder	1.1556	-3.39E-01	0.0580	0.2531
Currently diagnosed with bipolar disorder	0.8212	3.72E-01	0.0658	0.2225
Dietary Variables				
Years since meat consumption	2.3840	6.53E-01	0.2354	NA
Proportion of life spent vegetarian (grouped 0-2)	2.2759	6.36E-01	0.2354	NA
Years spent vegetarian (grouped 0-2)	2.0771	1.10E+00	0.2354	NA
Proportion of life spent vegetarian	2.9571	8.98E-01	0.2354	NA
Age when last ate meat	2.3560	-7.10E-01	0.2354	NA
Eats eggs	0.8976	1.36E-01	0.0011	0.7712
Eats dairy	2.1109	-1.11E+00	0.0247	0.1651
Eats wheat	1.0660	-3.85E-02	0.0000	0.9617
Eats sugar	1.1189	-9.99E-02	0.0016	0.7266

Table S9: Linear regression statistics for all TOPMed-imputed samples in individuals without a psychotic disorder.

TOPMed	Y-intercept	Beta coefficient	R^2 difference	P-value
Brain Region Volumes<br>Volume of gray matter within:				
Frontal pole (left)	0.8889	-5.35E-03	4.40E-05	0.2061
Frontal pole (right)	0.8901	-1.88E-03	5.45E-06	0.6564
Insular cortex (left)	0.8887	-5.59E-03	5.11E-05	0.1731
Insular cortex (right)	0.8880	-7.33E-03	8.78E-05	0.0742
Superior frontal gyrus (left)	0.8903	-2.35E-03	9.97E-06	0.5474
Superior frontal gyrus (right)	0.8902	-2.57E-03	1.20E-05	0.5091
Middle frontal gyrus (left)	0.8906	-9.28E-04	1.56E-06	0.8116
Middle frontal gyrus (right)	0.8903	-2.07E-03	7.74E-06	0.5960
Inferior frontal gyrus: pars triangularis (left)	0.8901	-5.40E-03	5.50E-05	0.1576
Inferior frontal gyrus: pars triangularis (right)	0.8903	-3.25E-03	1.98E-05	0.3965
Inferior frontal gyrus: pars opercularis (left)	0.8898	-6.45E-03	7.71E-05	0.0942
Inferior frontal gyrus: pars opercularis (right)	0.8905	-2.19E-03	9.04E-06	0.5665
Precentral gyrus (left)	0.8912	1.60E-03	4.34E-06	0.6912
Precentral gyrus (right)	0.8909	2.47E-04	1.03E-07	0.9512
Temporal pole (left)	0.8907	-3.20E-04	1.62E-07	0.9389
Temporal pole (right)	0.8915	1.99E-03	6.53E-06	0.6261
Anterior superior temporal gyrus (left)	0.8908	-8.69E-05	1.36E-08	0.9823
Anterior superior temporal gyrus (right)	0.8905	-1.26E-03	2.88E-06	0.7463
Posterior superior temporal gyrus (left)	0.8901	-2.95E-03	1.56E-05	0.4522
Posterior superior temporal gyrus (right)	0.8896	-4.92E-03	4.24E-05	0.2146
Anterior middle temporal gyrus (left)	0.8913	1.60E-03	4.45E-06	0.6878
Anterior middle temporal gyrus (right)	0.8913	2.12E-03	8.18E-06	0.5856
Posterior middle temporal gyrus (left)	0.8904	-1.73E-03	5.25E-06	0.6623
Posterior middle temporal gyrus (right)	0.8907	-6.47E-04	7.51E-07	0.8688
Middle temporal gyrus, temporo-occipital region (left)	0.8909	8.28E-04	1.32E-06	0.8268
Middle temporal gyrus, temporo-occipital region (right)	0.8896	-4.15E-03	3.06E-05	0.2918
Anterior inferior temporal gyrus (left)	0.8901	-2.59E-03	1.21E-05	0.5068
Anterior inferior temporal gyrus (right)	0.8904	-1.44E-03	3.75E-06	0.7121
Posterior inferior temporal gyrus (left)	0.8918	3.14E-03	1.75E-05	0.4249
Posterior inferior temporal gyrus (right)	0.8914	2.17E-03	8.51E-06	0.5783
Inferior temporal gyrus, temporo-occipital region (left)	0.8924	5.73E-03	5.98E-05	0.1404
Inferior temporal gyrus, temporo-occipital region (right)	0.8894	-4.07E-03	2.86E-05	0.3081
Postcentral gyrus (left)	0.8901	-5.32E-03	5.07E-05	0.1747
Postcentral gyrus (right)	0.8910	1.92E-03	6.60E-06	0.6244

Superior parietal lobule (left)	0.8917	5.99E-03	6.74E-05	0.1176
Superior parietal lobule (right)	0.8916	5.54E-03	5.83E-05	0.1457
Anterior supramarginal gyrus (left)	0.8908	-3.06E-04	1.79E-07	0.9357
Anterior supramarginal gyrus (right)	0.8909	1.09E-03	2.32E-06	0.7715
Posterior supramarginal gyrus (left)	0.8901	-3.41E-03	2.18E-05	0.3740
Posterior supramarginal gyrus (right)	0.8905	-1.51E-03	4.19E-06	0.6963
Angular gyrus (left)	0.8897	-6.41E-03	7.90E-05	0.0901
Angular gyrus (right)	0.8902	-3.06E-03	1.73E-05	0.4274
Superior lateral occipital cortex (left)	0.8915	3.82E-03	2.62E-05	0.3292
Superior lateral occipital cortex (right)	0.8906	-9.96E-04	1.72E-06	0.8027
Inferior lateral occipital cortex (left)	0.8911	1.34E-03	3.28E-06	0.7300
Inferior lateral occipital cortex (right)	0.8924	4.95E-03	4.17E-05	0.2182
Intracalcarine cortex (left)	0.8890	-7.81E-03	1.13E-04	0.0427
Intracalcarine cortex (right)	0.8892	-6.73E-03	8.36E-05	0.0813
Frontal medial cortex (left)	0.8914	2.52E-03	1.14E-05	0.5201
Frontal medial cortex (right)	0.8913	2.16E-03	8.48E-06	0.5788
Juxtapositional lobule cortex (left)	0.8906	-1.06E-03	2.08E-06	0.7834
Juxtapositional lobule cortex (right)	0.8900	-4.78E-03	4.25E-05	0.2138
Subcallosal cortex (left)	0.8906	-4.49E-04	3.32E-07	0.9126
Subcallosal cortex (right)	0.8907	-2.51E-04	1.04E-07	0.9510
Paracingulate gyrus (left)	0.8897	-4.40E-03	3.21E-05	0.2800
Paracingulate gyrus (right)	0.8902	-2.62E-03	1.15E-05	0.5187
Anterior cingulate gyrus (left)	0.8896	-5.28E-03	5.27E-05	0.1665
Anterior cingulate gyrus (right)	0.8888	-8.38E-03	1.32E-04	0.0285
Posterior cingulate gyrus (left)	0.8887	-5.05E-03	4.05E-05	0.2251
Posterior cingulate gyrus (right)	0.8878	-7.51E-03	9.17E-05	0.0680
Precuneous cortex (left)	0.8919	3.55E-03	2.11E-05	0.3810
Precuneous cortex (right)	0.8906	-6.52E-04	7.09E-07	0.8725
Cuneal cortex (left)	0.8906	-1.35E-03	3.37E-06	0.7264
Cuneal cortex (right)	0.8915	2.97E-03	1.57E-05	0.4495
Frontal orbital cortex (left)	0.8900	-2.16E-03	7.28E-06	0.6072
Frontal orbital cortex (right)	0.8906	-5.02E-04	4.00E-07	0.9041
Anterior parahippocampal gyrus (left)	0.8904	-1.04E-03	1.76E-06	0.8005
Anterior parahippocampal gyrus (right)	0.8900	-2.10E-03	7.06E-06	0.6126
Posterior parahippocampal gyrus (left)	0.8905	-1.37E-03	3.34E-06	0.7275
Posterior parahippocampal gyrus (right)	0.8905	-1.28E-03	2.98E-06	0.7420
Lingual gyrus (left)	0.8884	-5.28E-03	4.23E-05	0.2149
Lingual gyrus (right)	0.8883	-5.73E-03	5.03E-05	0.1765
Anterior temporal fusiform cortex (left)	0.8903	-1.58E-03	4.23E-06	0.6949
Anterior temporal fusiform cortex (right)	0.8888	-5.67E-03	5.34E-05	0.1635
Posterior temporal fusiform cortex (left)	0.8913	1.26E-03	2.57E-06	0.7602
Posterior temporal fusiform cortex (right)	0.8914	1.80E-03	5.36E-06	0.6592
Temporal occipital fusiform cortex (left)	0.8890	-6.43E-03	7.38E-05	0.1016
Temporal occipital fusiform cortex (right)	0.8896	-3.69E-03	2.33E-05	0.3576
Occipital fusiform gyrus (left)	0.8902	-2.20E-03	8.38E-06	0.5812

Occipital fusiform gyrus (right)	0.8902	-2.34E-03	9.46E-06	0.5577
Frontal operculum cortex (left)	0.8900	-3.62E-03	2.29E-05	0.3620
Frontal operculum cortex (right)	0.8894	-6.76E-03	8.13E-05	0.0857
Central opercular cortex (left)	0.8895	-4.78E-03	3.75E-05	0.2432
Central opercular cortex (right)	0.8897	-4.33E-03	3.16E-05	0.2841
Parietal operculum cortex (left)	0.8908	-7.99E-07	1.10E-12	0.9998
Parietal operculum cortex (right)	0.8894	-4.33E-03	3.22E-05	0.2794
Planum polare (left)	0.8887	-1.15E-02	2.29E-04	0.0040
Planum polare (right)	0.8895	-6.46E-03	6.96E-05	0.1119
Heschl's gyrus (left)	0.8898	-3.98E-03	2.64E-05	0.3275
Heschl's gyrus (right)	0.8899	-3.79E-03	2.36E-05	0.3544
Planum temporale (left)	0.8910	4.95E-04	4.03E-07	0.9037
Planum temporale (right)	0.8891	-4.81E-03	3.83E-05	0.2384
Supracalcarine cortex (left)	0.8892	-6.58E-03	8.03E-05	0.0876
Supracalcarine cortex (right)	0.8906	-7.63E-04	1.07E-06	0.8437
Occipital pole (left)	0.8908	-2.17E-04	8.10E-08	0.9567
Occipital pole (right)	0.8898	-2.85E-03	1.33E-05	0.4874
Thalamus (left)	0.8888	-7.29E-03	9.69E-05	0.0606
Thalamus (right)	0.8889	-6.02E-03	6.40E-05	0.1274
Caudate (left)	0.8885	-1.37E-02	3.60E-04	0.0003
Caudate (right)	0.8884	-1.20E-02	2.72E-04	0.0017
Putamen (left)	0.8897	-5.90E-03	6.72E-05	0.1181
Putamen (right)	0.8902	-3.03E-03	1.76E-05	0.4235
Pallidum (left)	0.8902	-1.40E-02	3.74E-04	0.0002
Pallidum (right)	0.8900	-1.16E-02	2.54E-04	0.0024
Hippocampus (left)	0.8898	-2.92E-03	1.40E-05	0.4754
Hippocampus (right)	0.8890	-5.45E-03	4.94E-05	0.1802
Amygdala (left)	0.8890	-5.59E-03	4.95E-05	0.1797
Amygdala (right)	0.8910	6.05E-04	5.82E-07	0.8844
Ventral striatum (left)	0.8900	-3.60E-03	2.10E-05	0.3820
Ventral striatum (right)	0.8905	-1.03E-03	1.73E-06	0.8022
Brainstem	0.8893	-6.39E-03	6.62E-05	0.1210
IIV cerebellum (left)	0.8915	3.44E-03	2.16E-05	0.3754
IIV cerebellum (right)	0.8910	8.20E-04	1.22E-06	0.8332
V cerebellum (left)	0.8909	3.75E-04	2.48E-07	0.9243
V cerebellum (right)	0.8910	6.88E-04	8.32E-07	0.8620
VI cerebellum (left)	0.8894	-6.29E-03	6.65E-05	0.1201
VI cerebellum (vermis)	0.8911	3.08E-03	1.78E-05	0.4209
VI cerebellum (right)	0.8899	-4.18E-03	2.97E-05	0.2992
Crus I cerebellum (left)	0.8907	-6.21E-04	6.72E-07	0.8759
Crus I cerebellum (vermis)	0.8909	2.37E-03	1.12E-05	0.5244
Crus I cerebellum (right)	0.8909	2.77E-04	1.29E-07	0.9454
Crus II cerebellum (left)	0.8905	-1.39E-03	3.52E-06	0.7206
Crus II cerebellum (vermis)	0.8912	6.04E-03	7.19E-05	0.1059
Crus II cerebellum (right)	0.8904	-2.10E-03	7.94E-06	0.5913

VIIb cerebellum (left)	0.8904	-2.46E-03	1.09E-05	0.5288
VIIb cerebellum (vermis)	0.8908	6.39E-04	7.79E-07	0.8664
VIIb cerebellum (right)	0.8908	-1.53E-04	4.23E-08	0.9687
VIIIa cerebellum (left)	0.8908	2.64E-04	1.23E-07	0.9467
VIIIa cerebellum (vermis)	0.8908	1.49E-04	4.12E-08	0.9692
VIIIa cerebellum (right)	0.8910	2.35E-03	9.82E-06	0.5504
VIIIb cerebellum (left)	0.8904	-2.41E-03	1.05E-05	0.5364
VIIIb cerebellum (vermis)	0.8910	1.89E-03	6.73E-06	0.6211
VIIIb cerebellum (right)	0.8907	-1.57E-03	4.46E-06	0.6873
IX cerebellum (left)	0.8908	-2.79E-04	1.49E-07	0.9413
IX cerebellum (vermis)	0.8909	1.37E-03	3.64E-06	0.7163
IX cerebellum (right)	0.8908	-1.56E-03	4.61E-06	0.6823
X cerebellum (left)	0.8913	1.82E-03	5.72E-06	0.6486
X cerebellum (vermis)	0.8908	6.88E-05	8.98E-09	0.9856
X cerebellum (right)	0.8931	9.84E-03	1.68E-04	0.0135
Psychological Variables				
Ever addicted to any substance or behaviour	0.8853	-1.20E-02	1.55E-05	0.5335
Lifetime number of depressed periods	0.8855	-3.35E-03	2.24E-05	0.5796
Ever taken cannabis	0.8841	-4.23E-03	3.40E-05	0.3537
Ever heard an unreal voice	0.8837	3.64E-02	4.33E-05	0.2959
Ever believed in an unreal conspiracy against self	0.8840	3.70E-02	1.66E-05	0.5169
Ever seen an unreal vision	0.8835	2.53E-02	3.80E-05	0.3290
Ever believed in unreal communications or signs	0.8843	-5.50E-02	3.52E-05	0.3456
Ever thought that life not worth living	0.8847	-7.01E-03	9.60E-05	0.1201
Ever self-harmed	0.8845	-5.39E-03	2.32E-06	0.8087
Ever attempted suicide	0.8576	2.56E-02	3.10E-04	0.5729
Ever sought or received professional help for mental distress	0.8835	2.02E-03	1.88E-06	0.8277
Ever diagnosed with IBS	0.8895	-2.67E-03	1.41E-06	0.8432
Ever been offered or sought treatment for anxiety	0.8907	-8.68E-03	2.52E-05	0.3980
Ever been offered or sought treatment for depression	0.8877	1.77E-03	1.11E-06	0.8590
Mother had depression	0.8897	1.85E-02	4.23E-05	0.2182
Father had depression	0.8917	-1.24E-02	1.04E-05	0.5435
Mother had Alzheimer's	0.8904	9.49E-03	1.17E-05	0.5167
Father had Alzheimer's	0.8915	-4.94E-03	1.70E-06	0.8057
Currently diagnosed with Major Depressive Disorder	0.8993	-2.32E-02	2.03E-04	0.1762
Currently diagnosed with bipolar disorder	0.8919	-6.86E-03	7.71E-07	0.9336
Dietary Variables				
Years since meat consumption	0.8668	-3.43E-02	2.35E-03	0.0393
Proportion of life spent vegetarian (grouped 0-2)	0.8636	-3.29E-02	2.21E-03	0.0456
Years spent vegetarian (grouped 0-2)	0.8656	-3.58E-02	2.64E-03	0.0288
Proportion of life spent vegetarian	0.8631	-2.90E-02	1.70E-03	0.0795

Age when last ate meat	0.8587	3.76E-02	2.35E-03	0.0393
Eats eggs	0.8647	2.65E-02	2.54E-05	0.3369
Eats dairy	0.8862	4.63E-03	4.33E-07	0.9002
Eats wheat	0.8212	7.09E-02	1.28E-04	0.0310
Eats sugar	0.8921	-1.46E-03	5.05E-07	0.8922

Table S10: Linear regression statistics for all 139 brain regions using total gray matter volume-adjusted regional volumes.

Brain Region	β coefficient	SE	95% CI	p-value	FDR-adjusted p	Bonferroni-adjusted p
Frontal pole (left)	−0.0054	0.0068	−0.0186 to 0.0079	0.4281	0.9025	1.0000
Frontal pole (right)	0.0029	0.0068	−0.0104 to 0.0162	0.6714	0.9431	1.0000
Insular cortex (left)	−0.0070	0.0058	−0.0184 to 0.0043	0.2247	0.8561	1.0000
Insular cortex (right)	−0.0106	0.0057	−0.0217 to 0.0006	0.0644	0.5966	1.0000
Superior frontal gyrus (left)	−0.0005	0.0047	−0.0097 to 0.0087	0.9127	0.9975	1.0000
Superior frontal gyrus (right)	−0.0013	0.0046	−0.0104 to 0.0078	0.7833	0.9783	1.0000
Middle frontal gyrus (left)	0.0012	0.0047	−0.0081 to 0.0104	0.8053	0.9783	1.0000
Middle frontal gyrus (right)	−0.0002	0.0048	−0.0096 to 0.0091	0.9612	0.9975	1.0000
Inferior frontal gyrus: pars triangularis (left)	−0.0048	0.0040	−0.0128 to 0.0031	0.2313	0.8561	1.0000
Inferior frontal gyrus: pars triangularis (right)	−0.0024	0.0040	−0.0103 to 0.0056	0.5584	0.9206	1.0000
Inferior frontal gyrus: pars opercularis (left)	−0.0059	0.0042	−0.0141 to 0.0022	0.1546	0.8561	1.0000
Inferior frontal gyrus: pars opercularis (right)	−0.0009	0.0041	−0.0089 to 0.0071	0.8273	0.9803	1.0000
Precentral gyrus (left)	0.0056	0.0051	−0.0044 to 0.0156	0.2696	0.8561	1.0000
Precentral gyrus (right)	0.0038	0.0051	−0.0062 to 0.0138	0.4545	0.9025	1.0000
Temporal pole (left)	0.0031	0.0050	−0.0066 to 0.0128	0.5334	0.9206	1.0000
Temporal pole (right)	0.0057	0.0049	−0.0038 to 0.0153	0.2412	0.8561	1.0000
Anterior superior temporal gyrus (left)	0.0016	0.0043	−0.0069 to 0.0101	0.7089	0.9431	1.0000
Anterior superior temporal gyrus (right)	−0.0000	0.0043	−0.0084 to 0.0084	0.9964	0.9975	1.0000
Posterior superior temporal gyrus (left)	−0.0016	0.0044	−0.0103 to 0.0070	0.7124	0.9431	1.0000
Posterior superior temporal gyrus (right)	−0.0038	0.0046	−0.0129 to 0.0052	0.4078	0.9025	1.0000
Anterior middle temporal gyrus (left)	0.0039	0.0044	−0.0047 to 0.0126	0.3698	0.9025	1.0000
Anterior middle temporal gyrus (right)	0.0040	0.0043	−0.0043 to 0.0124	0.3458	0.9025	1.0000
Posterior middle temporal gyrus (left)	−0.0000	0.0048	−0.0094 to 0.0093	0.9975	0.9975	1.0000
Posterior middle temporal gyrus (right)	0.0020	0.0047	−0.0073 to 0.0113	0.6797	0.9431	1.0000
Middle temporal gyrus, temporo-occipital region (left)	0.0022	0.0041	−0.0059 to 0.0102	0.5988	0.9249	1.0000

Middle temporal gyrus, temporo-occipital region (right)	−0.0036	0.0044	−0.0122 to 0.0050	0.4144	0.9025	1.0000
Anterior inferior temporal gyrus (left)	−0.0016	0.0041	−0.0096 to 0.0065	0.7028	0.9431	1.0000
Anterior inferior temporal gyrus (right)	−0.0002	0.0041	−0.0083 to 0.0079	0.9578	0.9975	1.0000
Posterior inferior temporal gyrus (left)	0.0058	0.0044	−0.0029 to 0.0144	0.1905	0.8561	1.0000
Posterior inferior temporal gyrus (right)	0.0051	0.0044	−0.0035 to 0.0137	0.2471	0.8561	1.0000
Inferior temporal gyrus, temporo-occipital region (left)	0.0080	0.0042	−0.0002 to 0.0162	0.0561	0.5565	1.0000
Inferior temporal gyrus, temporo-occipital region (right)	−0.0029	0.0044	−0.0115 to 0.0057	0.5126	0.9206	1.0000
Postcentral gyrus (left)	−0.0051	0.0048	−0.0145 to 0.0042	0.2829	0.8561	1.0000
Postcentral gyrus (right)	0.0054	0.0047	−0.0039 to 0.0147	0.2527	0.8561	1.0000
Superior parietal lobule (left)	0.0090	0.0042	0.0008 to 0.0172	0.0323	0.5565	1.0000
Superior parietal lobule (right)	0.0082	0.0041	0.0001 to 0.0162	0.0478	0.5565	1.0000
Anterior supramarginal gyrus (left)	0.0017	0.0042	−0.0064 to 0.0099	0.6742	0.9431	1.0000
Anterior supramarginal gyrus (right)	0.0031	0.0041	−0.0050 to 0.0112	0.4536	0.9025	1.0000
Posterior supramarginal gyrus (left)	−0.0020	0.0042	−0.0103 to 0.0064	0.6441	0.9431	1.0000
Posterior supramarginal gyrus (right)	0.0000	0.0042	−0.0082 to 0.0083	0.9937	0.9975	1.0000
Angular gyrus (left)	−0.0058	0.0041	−0.0139 to 0.0023	0.1628	0.8561	1.0000
Angular gyrus (right)	−0.0019	0.0042	−0.0102 to 0.0064	0.6523	0.9431	1.0000
Superior lateral occipital cortex (left)	0.0096	0.0050	−0.0002 to 0.0195	0.0559	0.5565	1.0000
Superior lateral occipital cortex (right)	0.0017	0.0050	−0.0081 to 0.0115	0.7342	0.9450	1.0000
Inferior lateral occipital cortex (left)	0.0039	0.0044	−0.0047 to 0.0126	0.3756	0.9025	1.0000
Inferior lateral occipital cortex (right)	0.0096	0.0047	0.0003 to 0.0189	0.0421	0.5565	1.0000
Intracalcarine cortex (left)	−0.0077	0.0042	−0.0159 to 0.0006	0.0691	0.6005	1.0000
Intracalcarine cortex (right)	−0.0065	0.0043	−0.0148 to 0.0019	0.1280	0.8561	1.0000

Frontal medial cortex (left)	0.0048	0.0043	−0.0036 to 0.0133	0.2598	0.8561	1.0000
Frontal medial cortex (right)	0.0040	0.0042	−0.0043 to 0.0123	0.3452	0.9025	1.0000
Juxtapositional lobule cortex (left)	0.0005	0.0042	−0.0078 to 0.0087	0.9120	0.9975	1.0000
Juxtapositional lobule cortex (right)	−0.0038	0.0042	−0.0120 to 0.0045	0.3704	0.9025	1.0000
Subcallosal cortex (left)	0.0031	0.0055	−0.0076 to 0.0138	0.5756	0.9206	1.0000
Subcallosal cortex (right)	0.0036	0.0053	−0.0068 to 0.0139	0.5019	0.9206	1.0000
Paracingulate gyrus (left)	−0.0032	0.0051	−0.0132 to 0.0068	0.5330	0.9206	1.0000
Paracingulate gyrus (right)	−0.0008	0.0050	−0.0106 to 0.0089	0.8644	0.9975	1.0000
Anterior cingulate gyrus (left)	−0.0049	0.0042	−0.0131 to 0.0034	0.2451	0.8561	1.0000
Anterior cingulate gyrus (right)	−0.0081	0.0042	−0.0163 to 0.0000	0.0513	0.5565	1.0000
Posterior cingulate gyrus (left)	−0.0041	0.0053	−0.0146 to 0.0064	0.4436	0.9025	1.0000
Posterior cingulate gyrus (right)	−0.0081	0.0052	−0.0183 to 0.0022	0.1235	0.8561	1.0000
Precuneous cortex (left)	0.0110	0.0055	0.0002 to 0.0219	0.0458	0.5565	1.0000
Precuneous cortex (right)	0.0031	0.0056	−0.0080 to 0.0141	0.5837	0.9220	1.0000
Cuneal cortex (left)	0.0002	0.0043	−0.0083 to 0.0087	0.9666	0.9975	1.0000
Cuneal cortex (right)	0.0064	0.0045	−0.0024 to 0.0153	0.1559	0.8561	1.0000
Frontal orbital cortex (left)	0.0004	0.0054	−0.0102 to 0.0110	0.9382	0.9975	1.0000
Frontal orbital cortex (right)	0.0030	0.0052	−0.0072 to 0.0133	0.5616	0.9206	1.0000
Anterior parahippocampal gyrus (left)	0.0016	0.0046	−0.0075 to 0.0106	0.7311	0.9450	1.0000
Anterior parahippocampal gyrus (right)	−0.0003	0.0047	−0.0094 to 0.0089	0.9556	0.9975	1.0000
Posterior parahippocampal gyrus (left)	−0.0002	0.0043	−0.0087 to 0.0083	0.9571	0.9975	1.0000
Posterior parahippocampal gyrus (right)	−0.0001	0.0043	−0.0085 to 0.0083	0.9767	0.9975	1.0000
Lingual gyrus (left)	−0.0041	0.0052	−0.0143 to 0.0061	0.4314	0.9025	1.0000
Lingual gyrus (right)	−0.0050	0.0053	−0.0155 to 0.0054	0.3437	0.9025	1.0000

Anterior temporal fusiform cortex (left)	0.0004	0.0045	−0.0084 to 0.0092	0.9309	0.9975	1.0000
Anterior temporal fusiform cortex (right)	−0.0050	0.0045	−0.0138 to 0.0038	0.2634	0.8561	1.0000
Posterior temporal fusiform cortex (left)	0.0051	0.0050	−0.0047 to 0.0149	0.3059	0.9025	1.0000
Posterior temporal fusiform cortex (right)	0.0053	0.0049	−0.0043 to 0.0148	0.2797	0.8561	1.0000
Temporal occipital fusiform cortex (left)	−0.0063	0.0044	−0.0150 to 0.0024	0.1540	0.8561	1.0000
Temporal occipital fusiform cortex (right)	−0.0028	0.0046	−0.0118 to 0.0062	0.5445	0.9206	1.0000
Occipital fusiform gyrus (left)	−0.0001	0.0046	−0.0092 to 0.0090	0.9786	0.9975	1.0000
Occipital fusiform gyrus (right)	−0.0007	0.0046	−0.0097 to 0.0082	0.8696	0.9975	1.0000
Frontal operculum cortex (left)	−0.0025	0.0044	−0.0111 to 0.0062	0.5762	0.9206	1.0000
Frontal operculum cortex (right)	−0.0066	0.0043	−0.0150 to 0.0018	0.1230	0.8561	1.0000
Central opercular cortex (left)	−0.0050	0.0053	−0.0154 to 0.0053	0.3412	0.9025	1.0000
Central opercular cortex (right)	−0.0039	0.0052	−0.0141 to 0.0063	0.4494	0.9025	1.0000
Parietal operculum cortex (left)	0.0020	0.0045	−0.0067 to 0.0108	0.6529	0.9431	1.0000
Parietal operculum cortex (right)	−0.0040	0.0045	−0.0129 to 0.0049	0.3761	0.9025	1.0000
Planum polare (left)	−0.0146	0.0049	−0.0241 to −0.0051	0.0027	0.0663	0.3756
Planum polare (right)	−0.0070	0.0051	−0.0170 to 0.0030	0.1692	0.8561	1.0000
Heschl's gyrus (left)	−0.0031	0.0046	−0.0121 to 0.0058	0.4956	0.9206	1.0000
Heschl's gyrus (right)	−0.0029	0.0048	−0.0124 to 0.0065	0.5408	0.9206	1.0000
Planum temporale (left)	0.0025	0.0045	−0.0063 to 0.0114	0.5723	0.9206	1.0000
Planum temporale (right)	−0.0040	0.0047	−0.0132 to 0.0052	0.3956	0.9025	1.0000
Supracalcarine cortex (left)	−0.0060	0.0043	−0.0144 to 0.0023	0.1559	0.8561	1.0000
Supracalcarine cortex (right)	0.0011	0.0043	−0.0074 to 0.0096	0.7943	0.9783	1.0000
Occipital pole (left)	0.0018	0.0046	−0.0073 to 0.0109	0.6980	0.9431	1.0000
Occipital pole (right)	−0.0012	0.0047	−0.0104 to 0.0079	0.7961	0.9783	1.0000
Thalamus (left)	−0.0069	0.0042	−0.0152 to 0.0014	0.1011	0.8270	1.0000
Thalamus (right)	−0.0054	0.0043	−0.0138 to 0.0030	0.2046	0.8561	1.0000
Caudate (left)	−0.0142	0.0040	−0.0220 to −0.0063	3.82E-04	0.0265	0.0530
Caudate (right)	−0.0125	0.0041	−0.0204 to −0.0045	0.0021	0.0663	0.2861
Putamen (left)	−0.0052	0.0039	−0.0127 to 0.0024	0.1800	0.8561	1.0000
Putamen (right)	−0.0022	0.0039	−0.0098 to 0.0054	0.5728	0.9206	1.0000

Pallidum (left)	−0.0143	0.0038	−0.0218 to −0.0069	1.59E-04	0.0220	0.0220
Pallidum (right)	−0.0119	0.0038	−0.0194 to −0.0044	0.0019	0.0663	0.2611
Hippocampus (left)	−0.0012	0.0049	−0.0108 to 0.0085	0.8105	0.9783	1.0000
Hippocampus (right)	−0.0053	0.0049	−0.0148 to 0.0043	0.2822	0.8561	1.0000
Amygdala (left)	−0.0045	0.0049	−0.0142 to 0.0052	0.3606	0.9025	1.0000
Amygdala (right)	0.0040	0.0051	−0.0060 to 0.0141	0.4331	0.9025	1.0000
Ventral striatum (left)	−0.0020	0.0045	−0.0107 to 0.0068	0.6568	0.9431	1.0000
Ventral striatum (right)	0.0014	0.0045	−0.0074 to 0.0101	0.7625	0.9675	1.0000
Brainstem	−0.0056	0.0045	−0.0144 to 0.0032	0.2123	0.8561	1.0000
IIV cerebellum (left)	0.0053	0.0041	−0.0027 to 0.0134	0.1924	0.8561	1.0000
IIV cerebellum (right)	0.0024	0.0041	−0.0057 to 0.0105	0.5641	0.9206	1.0000
V cerebellum (left)	0.0020	0.0043	−0.0063 to 0.0103	0.6370	0.9431	1.0000
V cerebellum (right)	0.0025	0.0043	−0.0059 to 0.0109	0.5618	0.9206	1.0000
VI cerebellum (left)	−0.0062	0.0047	−0.0153 to 0.0030	0.1867	0.8561	1.0000
VI cerebellum (vermis)	0.0049	0.0040	−0.0030 to 0.0128	0.2230	0.8561	1.0000
VI cerebellum (right)	−0.0036	0.0047	−0.0127 to 0.0055	0.4415	0.9025	1.0000
Crus I cerebellum (left)	0.0016	0.0045	−0.0071 to 0.0103	0.7197	0.9438	1.0000
Crus I cerebellum (vermis)	0.0029	0.0037	−0.0043 to 0.0102	0.4281	0.9025	1.0000
Crus I cerebellum (right)	0.0030	0.0046	−0.0060 to 0.0121	0.5071	0.9206	1.0000
Crus II cerebellum (left)	0.0005	0.0043	−0.0079 to 0.0090	0.9018	0.9975	1.0000
Crus II cerebellum (vermis)	0.0077	0.0038	0.0002 to 0.0153	0.0442	0.5565	1.0000
Crus II cerebellum (right)	−0.0004	0.0043	−0.0089 to 0.0080	0.9172	0.9975	1.0000
VIIb cerebellum (left)	−0.0009	0.0043	−0.0093 to 0.0075	0.8322	0.9803	1.0000
VIIb cerebellum (vermis)	0.0019	0.0040	−0.0059 to 0.0097	0.6319	0.9431	1.0000
VIIb cerebellum (right)	0.0019	0.0043	−0.0066 to 0.0103	0.6658	0.9431	1.0000
VIIIa cerebellum (left)	0.0023	0.0044	−0.0063 to 0.0110	0.5961	0.9249	1.0000
VIIIa cerebellum (vermis)	0.0016	0.0041	−0.0065 to 0.0096	0.7042	0.9431	1.0000
VIIIa cerebellum (right)	0.0047	0.0044	−0.0039 to 0.0132	0.2833	0.8561	1.0000
VIIIb cerebellum (left)	−0.0013	0.0042	−0.0096 to 0.0070	0.7656	0.9675	1.0000
VIIIb cerebellum (vermis)	0.0035	0.0041	−0.0045 to 0.0115	0.3907	0.9025	1.0000
VIIIb cerebellum (right)	−0.0003	0.0042	−0.0085 to 0.0080	0.9524	0.9975	1.0000
IX cerebellum (left)	0.0006	0.0040	−0.0073 to 0.0085	0.8806	0.9975	1.0000
IX cerebellum (vermis)	0.0024	0.0040	−0.0053 to 0.0102	0.5378	0.9206	1.0000
IX cerebellum (right)	−0.0007	0.0041	−0.0087 to 0.0072	0.8539	0.9974	1.0000
X cerebellum (left)	0.0038	0.0043	−0.0046 to 0.0122	0.3771	0.9025	1.0000
X cerebellum (vermis)	0.0009	0.0040	−0.0068 to 0.0087	0.8164	0.9783	1.0000
X cerebellum (right)	0.0126	0.0042	0.0043 to 0.0209	0.0029	0.0663	0.3976

REFERENCES

1. Marees, A.T.; de Kluiver, H.; Stringer, S.; Vorspan, F.; Curis, E.; Marie-Claire, C.; Derks, E.M. A tutorial on conducting genome-wide association studies: Quality control and statistical analysis. *Int J Methods Psychiatr Res* **2018**, *27*, e1608, doi:10.1002/mpr.1608.
2. Rayner, W. HRC Imputation Preparation and Checking Tool. *McCarthy Group Tools, V4.3.0* **2017**.
3. Das, S.; Forer, L.; Schonherr, S.; Sidore, C.; Locke, A.E.; Kwong, A.; Vrieze, S.I.; Chew, E.Y.; Levy, S.; McGue, M.; et al. Next-generation genotype imputation service and methods. *Nat Genet* **2016**, *48*, 1284-1287, doi:10.1038/ng.3656.
4. Fuchsberger, C.; Abecasis, G.R.; Hinds, D.A. minimac2: faster genotype imputation. *Bioinformatics* **2015**, *31*, 782-4, doi:10.1093/bioinformatics/btu704.
5. Taliun, D.; Harris, D.N.; Kessler, M.D.; Carlson, J.; Szpiech, Z.A.; Torres, R.; Taliun, S.A.G.; Corvelo, A.; Gogarten, S.M.; Kang, H.M.; et al. Sequencing of 53,831 diverse genomes from the NHLBI TOPMed Program. *Nature* **2021**, *590*, 290-299, doi:10.1038/s41586-021-03205-y.
6. Du, Z.; Guo, S.; Sun, Y.; Zhou, Q.; Jiang, Y.; Shen, Y.; Zhu, H.; Zhou, Z.; Zhou, H. Causal relationships between dietary habits and five major mental disorders: A two-sample Mendelian randomization study. *J. Affect. Disord.* **2023**, *340*, 607-615, doi:10.1016/j.jad.2023.08.098.
7. Zhuo, C.; Zhu, J.; Wang, C.; Qu, H.; Ma, X.; Tian, H.; Liu, M.; Qin, W. Brain structural and functional dissociated patterns in schizophrenia. *BMC Psychiatry* **2017**, *17*, 45, doi:10.1186/s12888-017-1194-5.
8. van Erp, T.G.M.; Walton, E.; Hibar, D.P.; Schmaal, L.; Jiang, W.; Glahn, D.C.; Pearlson, G.D.; Yao, N.; Fukunaga, M.; Hashimoto, R.; et al. Cortical Brain Abnormalities in 4474 Individuals With Schizophrenia and 5098 Control Subjects via the Enhancing Neuro Imaging Genetics Through Meta Analysis (ENIGMA) Consortium. *Biol Psychiatry* **2018**, *84*, 644-654, doi:10.1016/j.biopsych.2018.04.023.
9. Curtis, M.T.; Coffman, B.A.; Salisbury, D.F. Parahippocampal area three gray matter is reduced in first-episode schizophrenia spectrum: Discovery and replication samples. *Hum Brain Mapp* **2021**, *42*, 724-736, doi:10.1002/hbm.25256.
10. Cohen, J. *Statistical Power Analysis for the Behavioral Sciences*, 2nd ed.; Lawrence Erlbaum Associates: Hillsdale, NJ, USA, 1988.
